# Effects of Vitamin D Supplementation on Glucose and Insulin Homeostasis and Incident Diabetes among Nondiabetic Adults: A Meta-Analysis of Randomized Controlled Trials

**DOI:** 10.1155/2018/7908764

**Published:** 2018-12-03

**Authors:** Huilin Tang, Deming Li, Yufeng Li, Xi Zhang, Yiqing Song, Xinli Li

**Affiliations:** ^1^Department of Epidemiology, Richard M. Fairbanks School of Public Health, Indiana University, Indianapolis, Indiana, USA; ^2^Center for Pharmacoepidemiology, Richard M. Fairbanks School of Public Health, Indiana University, Indianapolis, Indiana, USA; ^3^School of Public Health, Medical College of Soochow University, Suzhou, Jiangsu, China; ^4^Department of Endocrinology, Beijing Pinggu Hospital, Beijing, China; ^5^Clinical Research Unit, Xinhua Hospital Affiliated to Shanghai Jiaotong University School of Medicine, Shanghai, China; ^6^Jiangsu Key Laboratory of Preventive and Translational Medicine for Geriatric Diseases, School of Public Health, Soochow University, Suzhou, Jiangsu, China

## Abstract

**Aims:**

Emerging evidence has suggested a mechanistic link from vitamin D metabolism to glucose and insulin homeostasis. This study is aimed at specifically quantifying the direct effects of vitamin D supplementation on indexes of glucose and insulin homeostasis as well as incidence of type 2 diabetes (T2D) among nondiabetic adults.

**Methods:**

We systematically searched randomized controlled trials (RCTs) of vitamin D supplementation in nondiabetic adults in PubMed, EMBASE, and CENTRAL. Random-effects meta-analysis was conducted to pool the estimates.

**Results:**

Our meta-analysis included 47 RCTs involving 44,161 nondiabetic individuals with a median trial duration of 4 months and a median dose of 4000 IU/d. Vitamin D supplementation significantly reduced fasting glucose by 0.11 mmol/L, fasting insulin by 1.47 mIU/L, and HOMA-IR by 0.32 while increasing total 25 (OH) D levels by 40.14 nmol/L. We found no significant effects of vitamin D supplementation on insulin secretion or beta cell function indexes. Based on the data from six trials involving 39,633 participants and 2533 incident T2D cases, vitamin D supplementation was not associated with the risk of incident diabetes compared to placebo (pooled relative risk: 1.01, 95% confidence interval: 0.93 to 1.08).

**Conclusions:**

Our meta-analysis found that vitamin D supplementation might improve glucose and insulin metabolism without affecting the risk of T2D among nondiabetic adults.

## 1. Introduction

Because type 2 diabetes (T2D) has become an important public health problem worldwide, its prevention has become imperative [1, 2]. Over the past decade, a large body of evidence from both observational and experimental studies has clearly suggested vitamin D's nonskeletal effects, especially those on individual or combined metabolic syndrome parameters such as adiposity, blood pressure, lipid metabolism, glucose intolerance, insulin resistance and secretion, and other metabolic abnormalities [2–5]. Epidemiological studies have linked low vitamin D levels to the pathogenesis of diabetes [6] and also supported the favorable effects of adequate vitamin D intake on reducing the risk of T2D [7, 8]. Experimental studies have provided evidence for the direct beneficial effects of vitamin D supplementation on glucose and insulin homeostasis as well as other metabolic abnormalities in patients with diabetes [9–11]. However, those trials focused mainly on the treatment or adjuvant therapy effects of vitamin D on the progression of diabetes rather than on T2D onset.

Several studies have assessed associations between vitamin D and serum indexes of pancreatic *β*-cell function and insulin resistance that reflect the pathogenesis of T2D, including the quantitative insulin sensitivity check index (QUICKI), fasting insulin, and HbA1c [12, 13]. Some but not all trials have identified beneficial effects on insulin secretion, glucose homeostasis, and insulin resistance among nondiabetic individuals [14–19]. However, these trials are limited by small sample size, short intervention period, nonrandomized treatment allocation, and the lack of objective assessment of vitamin D status and insulin or glucose homeostasis [14–19]. In particular, few trials have focused mainly on the effects of vitamin D on the primary prevention of T2D. Although two systematic review and meta-analyses have been conducted to address the effects of vitamin D supplementation on glucose homeostasis and insulin resistance [10, 20], the results remain uncertain due to the inclusion of 10 or fewer randomized controlled trials (RCTs). Given the strong scientific premise and emerging evidence from new trials, there is a need for a meta-analysis to maximize statistical power to reliably quantify the direct effects of vitamin D supplementation on glucose and insulin homeostasis and incidence of T2D among nondiabetic adults.

## 2. Methods

### 2.1. Search Strategy

PubMed database, EMBASE, and Cochrane Central Register of Controlled Trials (CENTRAL) were searched for relevant articles from inception to November 21, 2017. The following search terms were used: (“vitamin D” OR “vitamin D2” OR “vitamin D3” OR cholecalciferol OR ergocalciferol OR alphacalcidol OR alfacalcidol OR calcitriol OR paricalcitol OR doxerocalciferol OR “25 (OH)D”) AND (pre-diabet∗ OR diabet∗ OR T2DM OR TIIDM or “Type 2 DM” OR “Type II DM” OR insulin OR Homa∗ OR OGTT OR “impaired fasting glucose” OR IFG OR “fasting plasma glucose” OR FPG OR hba1c OR “beta cell”) AND (random∗ or RCT). No restrictions on language were imposed. In addition, the reference lists of retrieved papers and recent reviews were screened.

### 2.2. Study Selection and Outcomes of Interest

Studies were included if they met the following criteria: (1) randomized controlled clinical trials, (2) adults (≥18 years) without diabetes, (3) oral vitamin D (vitamin D2 or vitamin D3) supplementation with or without calcium, (4) placebo or no treatment, with or without calcium as a comparator, and (5) reporting at least one of our outcomes of interest. Specifically, the primary outcomes were incidence of diabetes and changes in blood 25-hydroxy vitamin D (25 (OH) D) levels between baseline and posttrial. The secondary outcomes included indexes of glucose intolerance, insulin resistance, and insulin secretion, such as HbA1c (%), fasting glucose, fasting insulin, insulin sensitivity (QUICKI, homeostatic model assessment-insulin resistance (HOMA-IR, HOMA2-IR), homeostasis model assessment of insulin sensitivity (HOMA2%S)), *β*-cell function (homeostasis model assessment of *β*-cell function (HOMA-B, HOMA-%B, or HOMA2-%B)), and disposition index (DI). Studies of participants with diabetes, women with gestational diabetes mellitus, and patients with cancer, cardiovascular disease, chronic kidney disease, or undergoing dialysis were excluded.

### 2.3. Data Extraction and Quality Assessment

The following data were extracted from the studies included using a standardized data collection form: first author, publication year, study location, and basic characteristic information of participants, including the total number, age, sex, BMI, and healthy status. Baseline level of serum 25 (OH) D, the dose and type of vitamin D supplementation, choice of control, duration of follow-up, and outcomes were also extracted.

The Cochrane Collaboration's risk-of-bias method was used to assess the methodological quality of random sequence generation, allocation concealment, blinding methods, handling of incomplete outcome data, and selective reporting. Each domain was rated as low, unclear, or high. Two blinded authors performed the literature search, study selection, data extraction, and quality assessment. Disagreements were resolved by consensus.

### 2.4. Statistical Analysis

We performed meta-analyses using Der Simonian and Laird's random-effects model with inverse-variance (standard error) weighting of individual study results when data could be combined. The pooled risk ratios (RR) with their 95% confidence intervals (CIs) were calculated for diabetes risk as a binary outcome, and the pooled weighted mean differences (WMDs) with their 95% CIs were calculated for biomarkers as continuous variables. Between-study heterogeneity was calculated using *I* ^2^ statistics. The percentage of *I* ^2^ around 25% (*I* ^2^ = 25), 50% (*I* ^2^ = 50), and 75% (*I* ^2^ = 75) indicates low, medium, and high heterogeneity, respectively. To explore potential effect modifiers, we performed subgroup analyses stratified by vitamin D dose (≤1000 IU/d vs. >1000 and ≤4000 IU/d vs. >4000 IU/d) and trial duration (<3 months vs. ≥3 and <12 months vs. ≥12 months). Furthermore, we tested for a possible nonlinear dose- and time-response effect of vitamin D supplementation on each biomarker using restricted cubic spline regression analyses. For primary outcomes, a visual inspection of funnel plot and Egger's test were performed to explore potential publication bias. All analyses were performed using STATA software (version 12.0, StataCorp, College Station, TX). All statistical significance was defined as two-sided (*α* < 0.05) unless specified otherwise.

## 3. Results

### 3.1. Study Selection and Characteristics

**Figure 1:**
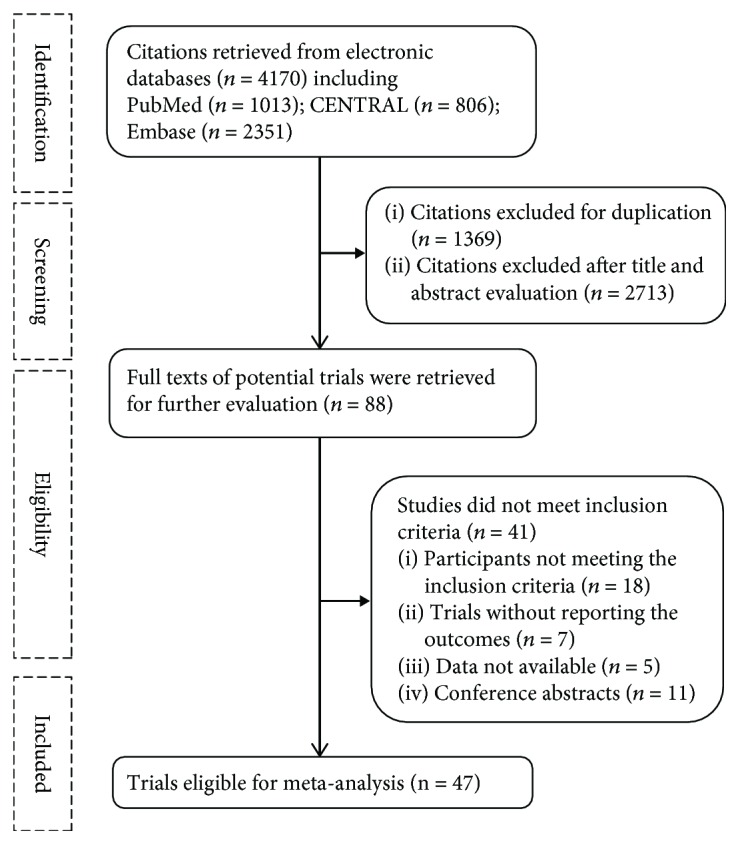
Flow chart of study selection.

The literature selection process is shown in Figure 1. Of the 4170 citations retrieved from electronic databases, 47 articles were included in our meta-analysis. The characteristics of the 47 RCTs are shown in Supplementary Table 1. A total of 44,161 participants (range: 12 to 33,951 individuals) were randomly assigned to vitamin D supplementation or placebo/no-treatment groups. Their ages ranged from 20.2 to 77 years and their BMI from 22.1 to 35.6 kg/m^2^. The mean or median baseline level of serum 25 (OH) D varied from 13.6 to 61.2 nmol/L. The participants received a median dose of 4000 IU/d (range: 125 to 12,695 IU/d; interquartile range: 1000 to 7142 IU/d) with a median duration of 4 months (range: 1 month to 84 months; interquartile range: 2.75 to 12 months). The risk of bias of the included trials is presented in Supplementary Figure 1. Most RCTs (>75%) displayed low risk of bias in terms of incomplete outcome data and selective reporting. However, the extent of random sequence generation, allocation concealment, or blinding methods was unclear or at high risk of bias for one-third to half of the included RCTs.

### 3.2. Elevated Changes in Serum 25 (OH) D Levels

Based on the data from 40 trials, vitamin D supplementation resulted in an increase in mean serum vitamin D levels by 41.06 nmol/L (41.16 nmol/L at baseline versus 82.22 nmol/L posttreatment) among participants taking vitamin D supplementation (Supplementary Figure 2). Moreover, our meta-analysis showed that vitamin D supplementation significantly increased serum 25 (OH) D levels by 40.14 nmol/L (95% CI: 37.07 to 43.22 nmol/L) compared to placebo (Table 1 and Supplementary Figure 3). Our stratified analysis by dose showed a linear trend towards a dose-response effect of vitamin D supplementation on the levels of serum 25 (OH) D (*p* for trend = 0.02) (Table 1). There was no significant difference among the subgroups stratified by duration (*p* for trend = 0.70) (Supplementary Table 2). Some evidence of publication bias was detected based on Egger's test (*p* = 0.01) and visual inspection of funnel plot (Supplementary Figure 4B). There was high heterogeneity in the meta-analyses (*I* ^2^ > 75%).

### 3.3. Changes in Indexes of Glucose Metabolism

As presented in Table 1 and Supplementary Table 2, vitamin D supplementation significantly decreased fasting insulin by 1.47 mIU/L (95% CI: −2.00 to −0.95) (Supplementary Figure 5) and fasting glucose by 0.11 mmol/L (95% CI: −0.17 to −0.04) (Supplementary Figure 6) compared to placebo. Our further subgroup analysis showed that vitamin D supplementation might decrease fasting insulin levels in a dose- (*p* for trend = 0.08) and duration-response manner (*p* for trend = 0.08). There were no significant differences between vitamin D supplementation and placebo groups in terms of changes in HbA1c (WMD: −0.04%, 95% CI: −0.07 to 0.00%) and 2 h glucose (WMD: −0.06 mmol/L, 95% CI: −0.47 to 0.35 mmol/L).

### 3.4. Changes in Insulin Sensitivity/Resistance Indexes

Overall, vitamin D supplementation was significantly associated with a reduction in HOMA-IR (WMD: −0.32, 95% CI: −0.47 to −0.17) compared with placebo (Table 1 and Supplementary Figure 7). Furthermore, a decrease in HOMA-IR remained significant with vitamin D supplementation >4000 IU/d (WMD: −0.45, 95% CI: −0.90 to −0.01) or longer than 3 months (WMD: −0.43, 95% CI: −0.60 to −0.26 for 3–12 months; WMD: −0.20, 95% CI: −0.36 to −0.03 for ≥12 months) (Table 1 and Supplementary Table 2). No significant differences in QUICKI, HOMA2-IR, and HOMA2-%S were observed between groups. However, high heterogeneity between studies was detected in our analysis (all *I* ^2^ > 75%).

In addition, there were no significant differences in 2 h insulin levels between vitamin D supplementation and placebo groups (WMD: −2.17 mIU/L, 95% CI: −15.60 to 11.25 mIU/L), although 2 h insulin was significantly higher in group receiving vitamin D supplementation (1000 IU/d to 4000 IU/d) compared with placebo (WMD: 5.70 mIU/L, 95% CI: 4.92 to 6.47 mIU/L).

### 3.5. Changes in Insulin Secretion/*β*-Cell Function Indexes

Supplementation of vitamin D decreased HOMA-B compared to placebo (WMD: −10.69, 95% CI: −19.10 to −2.29) (Table 1). Further stratified analyses found a significant decrease in HOMA-B in the subgroups receiving vitamin D supplementation 1000~4000 IU/d (WMD: −14.80, 95% CI: −24.76 to −4.84) and >4000 IU/d (WMD: −9.88, 95% CI: −19.69 to −0.07). There was no significant difference between vitamin D supplementation and placebo in terms of HOMA-%B, HOMA2-%B, or DI (Table 1 and Supplementary Table 2).

### 3.6. Dose-Response Analysis

To examine the nonlinear trend between vitamin D supplementation and serum 25 (OH) D, we included 40 trials and found a significant association between vitamin D supplementation and serum 25 (OH) D (*p* for nonlinearity = 0.01) (Supplementary Figure 8A). Serum 25 (OH) D increased continuously and then reached a plateau at about 4000 IU/d. We observed an abrupt increase in serum 25 (OH) D in the first 6 months of vitamin D supplementation but without a significant association (Supplementary Figure 9A). Vitamin D supplementation of about 4000 IU/day would be sufficient to decrease HbA1c, fasting glucose, and insulin and increase QUICKI (Supplementary Figure 8). We found an increase in QUICKI in the first 6 months of vitamin D supplementation (*p* for nonlinearity = 0.03) (Supplementary Figure 9E).

### 3.7. Effect on the Incidence of T2D

**Figure 2:**
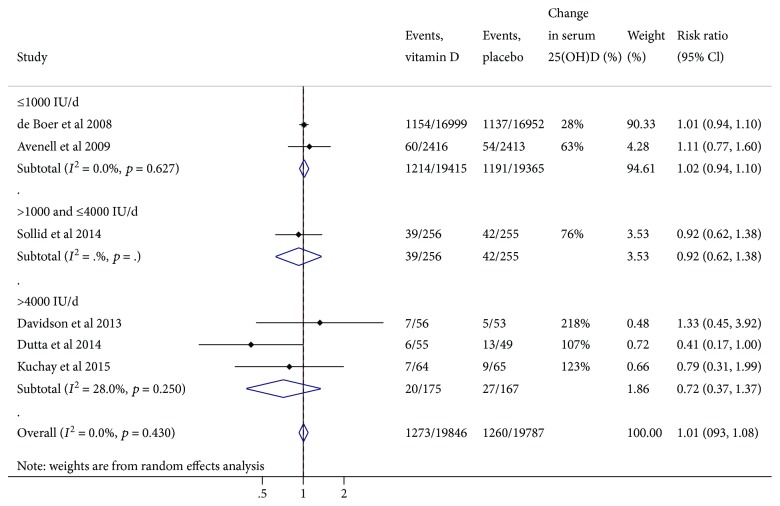
Meta-analysis of vitamin D supplementation on incidence of diabetes, stratified by dose.

Six trials encompassing 39,633 participants and 2533 incident T2D cases were included [19, 21–25]. Our meta-analysis found that vitamin D supplementation (median dose of 5714 and median duration of 12 months) had no significant effect on the risk of T2D (RR: 1.01, 95% CI: 0.93 to 1.08) (Figure 2). Our subgroup analysis showed that high-dose vitamin D supplementation (>4000 IU/d) did not decrease the risk of T2D (RR: 0.72, 95% CI: 0.37 to 1.37). No evidence of publication bias was detected based on Egger's test (*p* = 0.44) and visual inspection of funnel plot (Supplementary Figure 4A). One trial that evaluated the association between vitamin D supplementation and risk of diabetes or impaired fasting glucose among adults found that 700 IU/d vitamin D supplementation for 3 years did not prevent the development of diabetes or impaired fasting glucose [26]. Of the 7 trials involving participants with prediabetes [16, 17, 19, 21, 22, 25, 27], four trials [19, 21, 22, 25] and three trials [21, 22, 25] reported the outcomes of progression of prediabetes to diabetes and its reversal from prediabetes to normoglycemia, respectively. Vitamin D supplementation was not significantly associated with the increased risk of prediabetes progression to diabetes (RR, 0.83; 95% CI, 0.57 to 1.20) or its reversal to normoglycemia (RR, 1.20; 95% CI, 0.83 to 1.74) (Supplementary Figure 10); however, additional trials are needed to achieve the necessary power to address such impact.

## 4. Discussion

Our meta-analysis of RCTs found that vitamin D supplementation had no significant effect on incident diabetes. Vitamin D supplementation significantly decreased fasting levels of glucose, insulin, and HOMA-IR. Overall, our study provided evidence suggesting that vitamin D supplementation with a dose > 4000 IU/d may be sufficient to improve glucose and insulin homeostasis indices among nondiabetic participants. It should be noted that we found a decrease rather than an increase in HOMA-B, which should be considered an index of beta-cell “function” rather than “activity” [28]. Given the significant heterogeneity and relatively small sample sizes in available RCTs, additional large-scale and long-term randomized controlled trials in nondiabetic participants with vitamin D insufficiency are warranted to assess the efficacy of vitamin D supplementation on the primary prevention of T2D.

Several potential mechanisms have been proposed to explain the possible role of vitamin D supplementation in regulating the metabolism of glucose and insulin. A large body of literature has suggested that optimal vitamin D homeostasis is essential for both insulin sensitivity and secretion, which are fundamental to the pathogenesis of T2D. The widely accepted view was that vitamin D directly stimulates insulin receptors to enhance insulin sensitivity and insulin responsiveness for glucose transport [29, 30]. The effect of vitamin D on insulin resistance may be indirect, e.g., through beneficial effects on adiposity. Another explanation has elucidated that vitamin D affects the release of insulin via binding to the vitamin D receptor in *β*-cells and vitamin D-dependent calcium-binding proteins [31, 32]. Vitamin D may also indirectly affect calcium-dependent insulin secretion via regulation of calcium transport through *β*-cells [33]. However, the exact mechanisms remain unclear [30].

A majority of studies have investigated the association between vitamin D and glucose and insulin homeostasis among the patients with diabetes, while few studies have paid attention to this association among nondiabetic adults. One meta-analysis based on RCT data in nondiabetic populations suggested that vitamin D supplementation affected only fasting glucose in a subgroup with mean baseline HbA1c ≥ 8% but had no significant effect on HbA1c or HOMA-IR [9]. Similar to our present results, another meta-analysis showed that vitamin D had no effect on HOMA-IR but decreased fasting glucose and the level of HbA1c in prediabetes [10]. Moreover, we found a significant decrease in fasting insulin among the participants taking vitamin D supplementation especially among those taking a high dose (≥4000 IU/d), which suggests that a high dose of vitamin D supplementation might improve the insulin resistance. However, it should be noted that a greater reduction in fasting insulin was observed in the trials with shorter follow-up (<3 months) than those with longer follow-up (≥12 months), which might be a chance finding.

Vitamin D supplementation elevated the levels of serum 25 (OH) D and ameliorated the deficiency (or even normalized the levels) of vitamin D (serum 25 (OH) D < 30 nmol/L). Although some studies had shown that a dose of 10,000 IU/d vitamin D improved endothelial function and blood pressure [34–36], the recommended dietary allowances (RDAs) of vitamin D were 600 IU/d for ages 1~70 years and 800 IU/d for ages 71 years and older, corresponding to a serum 25 (OH) D level of at least 20 ng/mL (50 nmol/liter) according to a report from the Institute of Medicine (IOM) [37]. Among the RCTs included in our study, the intervention dose of vitamin D was over the RDA. In addition, some experts thought that the prevalence of vitamin D inadequacy had been overestimated [37] and suggested that long-term vitamin D supplementation might have an adverse effect on health [38]. It should be noted that current recommendations for vitamin D supplementation (200–600 IU/d) are inadequate to achieve optimal serum 25 (OH) D levels (>90 nmol/L). Thus, there is not yet any consensus as to whether the general population needs further supplementation of vitamin D, and a uniform guideline for vitamin D supplementation is still an issue worthy of discussion. However, it is widely accepted that patients with vitamin D deficiency should supplement vitamin D intake [37, 39]. Our subgroup analysis showed that vitamin D supplementation of 4000 IU/day might decrease fasting glucose, insulin, and HOMA-IR and increase QUICKI, which supports the beneficial effects of high-dose vitamin D supplementation on improving glucose metabolism.

In a review of studies of serum 25 (OH) D in relation to bone mineral density, lower extremity function, dental health, and risk of falls, fractures, and colorectal cancer, Boonen et al. found that optimal levels of serum 25 (OH) D were 90–100 nmol/L for all endpoints [40]. The average older individual requires an oral vitamin D_3_ intake of at least 800–1000 IU/d (20–25 *μ*g) to achieve a serum 25 (OH) D of 75 nmol/L [40]. The largest randomized trial, the Women's Health Initiative Clinical Trial of 33,951 initially nondiabetic postmenopausal women, did not observe any effect from daily intake of 1000 mg elemental calcium plus 400 IU vitamin D_3_ on risk of incident diabetes over 7 years of follow-up [23]. However, 400 IU vitamin D_3_ daily may have been too low to confer a clinical benefit. In particular, median levels of serum 25 (OH) D were raised from 42.3 to 54.1 nmol/L (roughly 12 nmol/L), which is lower than the optimal value of 75 nmol/L. Our meta-analysis found that serum 25 (OH) D was increased up to 82.22 nmol/L posttreatment among patients taking a median dose of 4000 IU/d vitamin D supplementation for a median duration of 3 months. Vitamin D supplementation > 4000 IU/d may be an option to improve glucose and insulin homeostasis indices among nondiabetic participants.

One main advantage of our meta-analysis was the inclusion of a large number of eligible RCTs, which enhanced its reliability and maximized statistical power. Another advantage was that we specifically assessed the dose-dependent effect of vitamin D supplementation. However, some limitations of our meta-analysis merit consideration. First, there was high between-trial heterogeneity in most subgroup analyses, which invalidated the effect estimates and limited the generalization of our findings to all populations. Second, the subjects of the RCTs included came from different countries and had different lifestyles and genetic backgrounds; such characteristics may modulate the effects of vitamin D supplementation. Third, available but limited RCT data revealed discernible effects of vitamin D supplementation on some but not all commonly used indices of glucose and insulin metabolisms with statistical significance. In particular, we lacked sufficient data to test the study hypothesis that vitamin D supplementation delays or even prevents the development of T2D among nondiabetic individuals. Finally, there was some evidence of publication bias in our meta-analysis. We cannot completely rule out the possibility that this affected the significance of our results.

## 5. Conclusions

In conclusion, our meta-analysis found no effect of vitamin D supplementation on incidence of T2D but suggested a possible dose-response effect of vitamin D supplementation on improving glucose and insulin metabolism among nondiabetic adults, indicating a possible benefit of taking high-dose vitamin D supplements for primary prevention of T2D. Future well-designed trials are warranted to confirm our findings and validate optimal vitamin D dosage.

## Figures and Tables

**Table 1:** Meta-analysis of vitamin D supplementation on indices of glucose and insulin homeostasis stratified by dose.

Outcomes	Overall			≤1000 IU/d			>1000 and ≤4000 IU/d			>4000 IU/d			*p* for trend
	*n*/*N*	WMD	*I* ^2^ (%)	*n*/*N*	WMD	*I* ^2^ (%)	*n*/*N*	WMD	*I* ^2^ (%)	*n*/*N*	WMD	*I* ^2^ (%)	
25 (OH) D	46/4992	40.14 (37.07, 43.22)	97.8	13/1661	31.16 (27.63, 34.09)	97.4	15/1603	39.64 (35.47, 43.82)	89.2	18/1728	47.34 (37.85, 56.84)	97.8	0.02
HbA1c	16/2298	−0.04 (−0.07, 0.00)	86.2	4/527	−0.07 (−0.16, 0.01)	88.7	7/1159	0.01 (−0.05, 0.07)	83.9	5/612	−0.11 (−0.26, 0.05)	84.1	0.97
Fasting glucose	40/5509	−0.11 (−0.17, −0.04)	96.1	12/2506	−0.08 (−0.18, 0.02)	97.8	14/1581	−0.08 (−0.14, −0.02)	46.9	14/1480	−0.16 (−0.38, 0.06)	94.5	0.42
Insulin	32/4740	−1.47 (−2.00, −0.95)	90.8	10/2192	−0.89 (−1.83, 0.05)	94.5	12/1381	−0.94 (−1.98, 0.11)	51.7	10/1167	−3.03 (−5.29, −0.76)	81.1	0.08
2 h glucose	15/1929	−0.06 (−0.47, 0.35)	88.4	3/171	0.13 (−0.21, 0.46)	0	8/1182	0.08 (−0.60, 0.77)	91.9	4/576	−0.47 (−1.34, 0.39)	89.3	0.24
2 h insulin	5/1379	−2.17 (−15.60, 11.25)	93.4	NA	NA	NA	3/870	5.70 (4.92, 6.47)	0	2/509	−13.29 (−39.69, 13.11)	94.0	0.17
*Insulin sensitivity*													
QUICKI	12/1740	0.00 (−0.00, 0.01)	72.1	1/123	−0.02 (−0.05, 0.01)	NA	4/919	0.00 (−0.00, 0.01)	75.2	7/698	0.01 (−0.00, 0.01)	73.9	0.55
HOMA-IR	36/5484	−0.32 (−0.47, −0.17)	96.1	13/2867	−0.24 (−0.50, 0.02)	97.0	9/1178	−0.22 (−0.54, 0.10)	67.3	14/1439	−0.45 (−0.90, −0.01)	95.9	0.34
HOMA2-IR	5/353	−0.14 (−0.31, 0.04)	46.6	NA	NA	NA	3/178	−0.21 (−0.46, 0.04)	42.1	2/175	−0.05 (−0.26, 0.16)	33.2	0.43
HOMA2-%S	5/329	0.57 (−3.24, 4.37)	22.6	NA	NA	NA	5/329	0.57 (−3.24, 4.37)	22.6	NA	NA	NA	NA
*Beta cell function*													
HOMA-B	6/404	−10.69 (−19.10, −2.29)	87.1	NA	NA	NA	1/70	−14.80 (−24.76, −4.84)	NA	5/334	−9.88 (−19.69, −0.07)	89.5	0.87
HOMA-%B	2/139	4.87 (−44.59, 54.34)	84.4	NA	NA	NA	NA	NA	NA	2/139	4.87 (−44.59, 54.34)	84.4	NA
HOMA2-%B	5/320	1.57 (−3.98, 7.12)	0	NA	NA	NA	4/249	0.28 (−5.61, 6.16)	0	1/71	12.03 (−4.70, 28.76)	NA	0.29
Disposition index	4/287	−0.05 (−0.39, 0.29)	1.9	NA	NA	NA	4/287	−0.05 (−0.39, 0.29)	1.9	NA	NA	NA	NA

*n*/*N*: number of studies/number of participants; 25 (OH) D: 25-hydroxyvitamin D; HOMA-IR: homeostatic model assessment-insulin resistance; QUICKI: quantitative insulin sensitivity check index; WMD: weighted mean difference; 2 h glucose: 2-hour plasma glucose; 2 h insulin: 2-hour plasma insulin; NA: not applicable.

## Data Availability

The data used to support the findings of this study are included within the article and the supplementary information file.

## Supplementary Materials

Supplementary Materials Supplementary Figure 1: risk of bias of included trials assessed by Cochrane risk of bias tool. Supplementary Figure 2: distributions of circulating 25 (OH) D levels in vitamin D group before and after taking vitamin D supplementation. Supplementary Figure 3: meta-analysis of vitamin D supplementation on serum 25 (OH) D levels stratified by dose. Supplementary Figure 4: a visual inspection of funnel plot of the effects of vitamin D supplementation on incidence of type 2 diabetes (A) and serum 25 (OH) D (B). Supplementary Figure 5: meta-analysis of vitamin D supplementation on fasting insulin stratified by dose. Supplementary Figure 6: meta-analysis of vitamin D supplementation on fasting glucose stratified by dose. Supplementary Figure 7: meta-analysis of vitamin D supplementation on HOMA-IR stratified by dose. Supplementary Figure 8: dose-response association between dose of vitamin D supplementation and change in serum 25 (OH) D levels (A), fasting glucose (B), insulin (C), HbA1c (D), QUICKI (E), and HOMA-IR (F) by using restricted cubic spline curves. The red lines and the gray shaded regions indicated the estimated value and 95% confidence interval. Supplementary Figure 9: dose-response association between the duration of vitamin D supplementation and change in serum 25 (OH) D levels (A), fasting glucose (B), insulin (C), HbA1c (D), QUICKI (E), and HOMA-IR (F) by using restricted cubic spline curves. The red lines and the gray shaded regions indicated the estimated value and 95% confidence interval. Supplementary Figure 10: meta-analysis of vitamin D supplementation on prediabetes progression to diabetes and its reversal to normoglycemia among participants with prediabetes. Supplementary Table 1: characteristics of included studies. Supplementary Table 2: meta-analysis of vitamin D supplementation on indexes of glucose and insulin homeostasis stratified by duration.
