# Supplementary material for: Effects of Vitamin D Supplementation on Glucose and Insulin Homeostasis and Incident Diabetes among Nondiabetic Adults: A Meta-Analysis of Randomized Controlled Trials
Source: Int J Endocrinol. 2018 Dec 3;2018:7908764. doi: 10.1155/2018/7908764 (PMC6304827; doi:10.1155/2018/7908764)
Supplement: Supplementary Materials — Supplementary Figure 1: risk of bias of included trials assessed by Cochrane risk of bias tool. Supplementary Figure 2: distributions of circulating 25 (OH) D levels in vitamin D group before and after taking vitamin D supplementation. Supplementary Figure 3: meta-analysis of vitamin D supplementation on serum 25 (OH) D levels stratified by dose. Supplementary Figure 4: a visual inspection of funnel plot of the effects of vitamin D supplementation on incidence of type 2 diabetes (A) and serum 25 (OH) D (B). Supplementary Figure 5: meta-analysis of vitamin D supplementation on fasting insulin stratified by dose. Supplementary Figure 6: meta-analysis of vitamin D supplementation on fasting glucose stratified by dose. Supplementary Figure 7: meta-analysis of vitamin D supplementation on HOMA-IR stratified by dose. Supplementary Figure 8: dose-response association between dose of vitamin D supplementation and change in serum 25 (OH) D levels (A), fasting glucose (B), insulin (C), HbA1c (D), QUICKI (E), and HOMA-IR (F) by using restricted cubic spline curves. The red lines and the gray shaded regions indicated the estimated value and 95% confidence interval. Supplementary Figure 9: dose-response association between the duration of vitamin D supplementation and change in serum 25 (OH) D levels (A), fasting glucose (B), insulin (C), HbA1c (D), QUICKI (E), and HOMA-IR (F) by using restricted cubic spline curves. The red lines and the gray shaded regions indicated the estimated value and 95% confidence interval. Supplementary Figure 10: meta-analysis of vitamin D supplementation on prediabetes progression to diabetes and its reversal to normoglycemia among participants with prediabetes. Supplementary Table 1: characteristics of included studies. Supplementary Table 2: meta-analysis of vitamin D supplementation on indexes of glucose and insulin homeostasis stratified by duration. [file 7908764.f1.docx]

**Supporting Information**

**Effects of vitamin D supplementation on glucose and insulin homeostasis and incident diabetes among non-diabetic adults: a systematic review and meta-analysis**

Huilin Tang, MSc^1,2*^; Deming Li, BSc^3*^, Yufeng Li, MD, PhD^4^, Xi Zhang, PhD^5^, Yiqing Song, MD, ScD^1,2^, Xinli Li, PhD^3,6^

^1^Department of Epidemiology, Richard M. Fairbanks School of Public Health, Indiana University, Indianapolis, Indiana, USA;

^2^Center for Pharmacoepidemiology, Richard M. Fairbanks School of Public Health, Indiana University, Indianapolis, Indiana, USA;

^3^School of Public Health, Medical College of Soochow University, Suzhou, Jiangsu, China;

^4^Department of Endocrinology, Beijing Pinggu Hospital, Beijing, China;

^5^Clinical Research Unit, Xinhua Hospital Affiliated to Shanghai Jiaotong University School of Medicine, Shanghai, China;

^6^Jiangsu Key Laboratory of Preventive and Translational Medicine for Geriatric Diseases, School of Public Health, Soochow University, Suzhou, Jiangsu, China.

**Supplementary Figure 1.** Risk of bias of included trials assessed by Cochrane risk of bias tool.


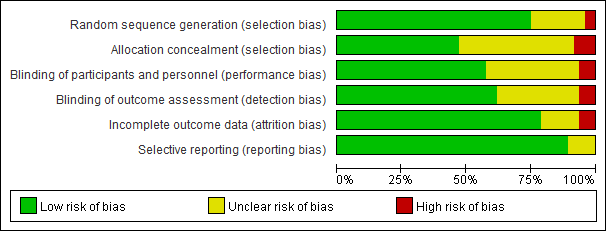


**Supplementary Figure 2.** Distributions of circulating 25(OH)D levels in vitamin D group before and after taking vitamin D supplementation.

**
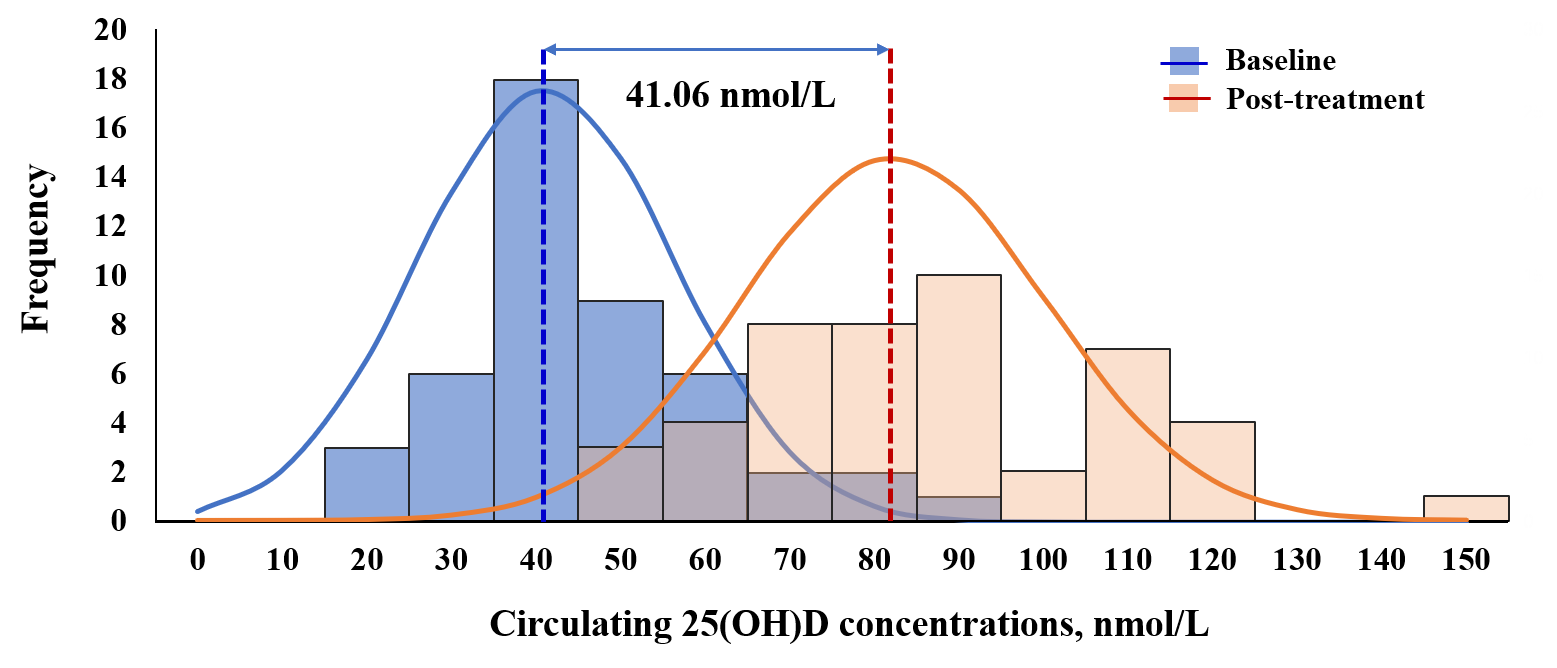
**

**Supplementary Figure 3. Meta-analysis of vitamin D supplementation on serum 25(OH)D levels stratified by dose.**


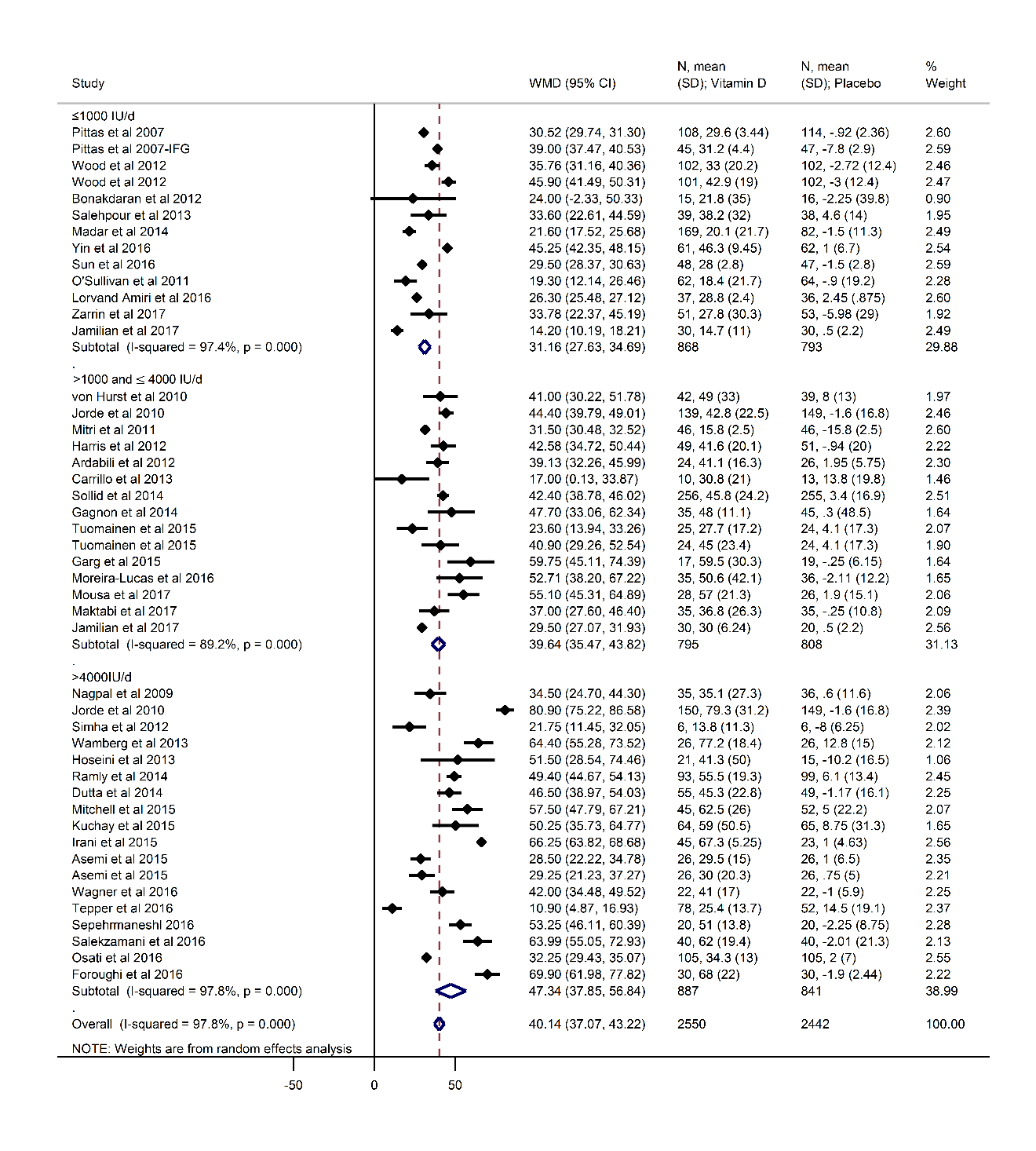


**Supplementary Figure 4**. A visual inspection of funnel plot of the effects of vitamin D supplementation on incidence of type 2 diabetes (A) and serum 25(OH)D (B).


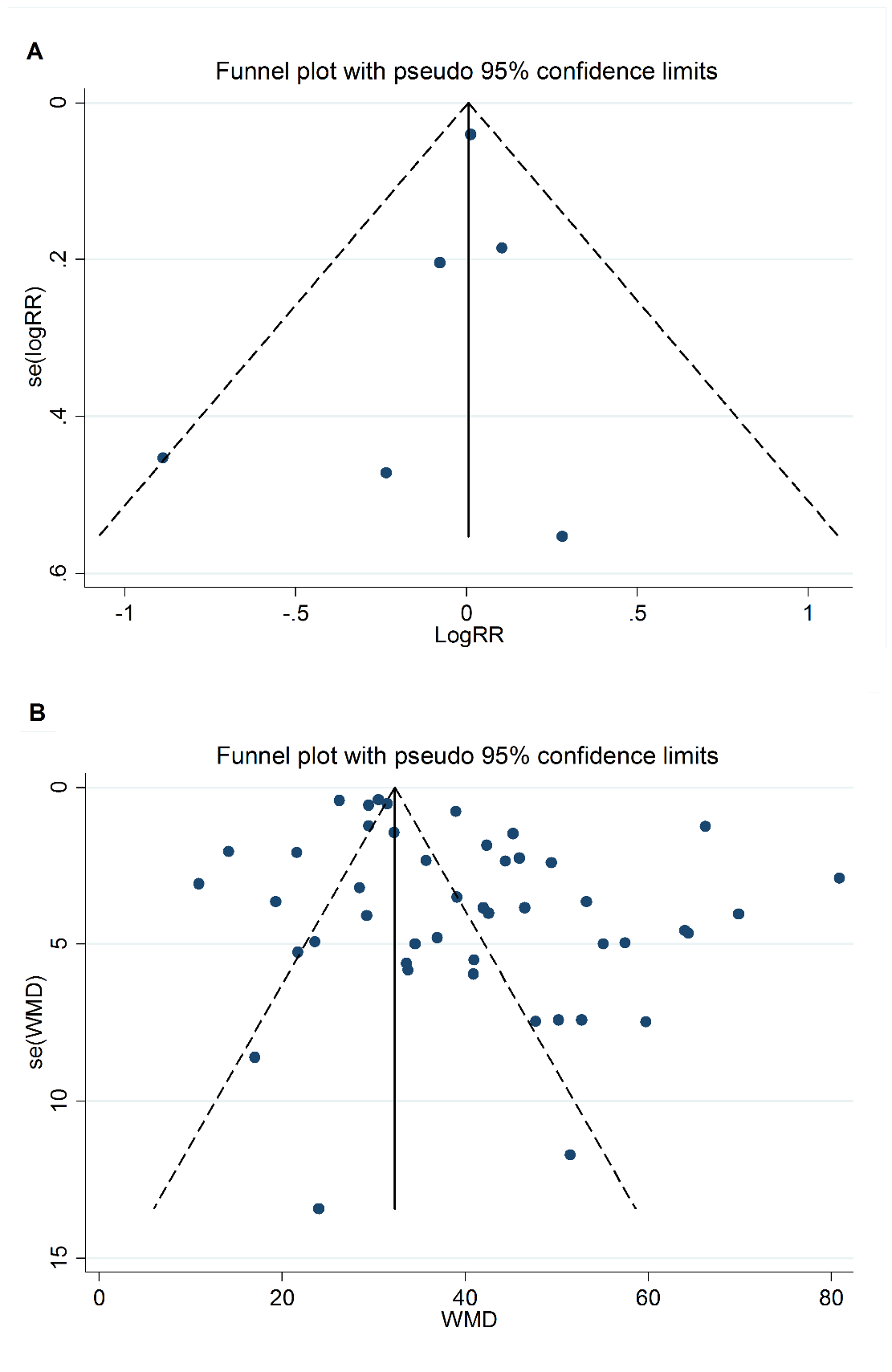


**Supplementary Figure 5. Meta-analysis of vitamin D supplementation on fasting insulin stratified by dose.**


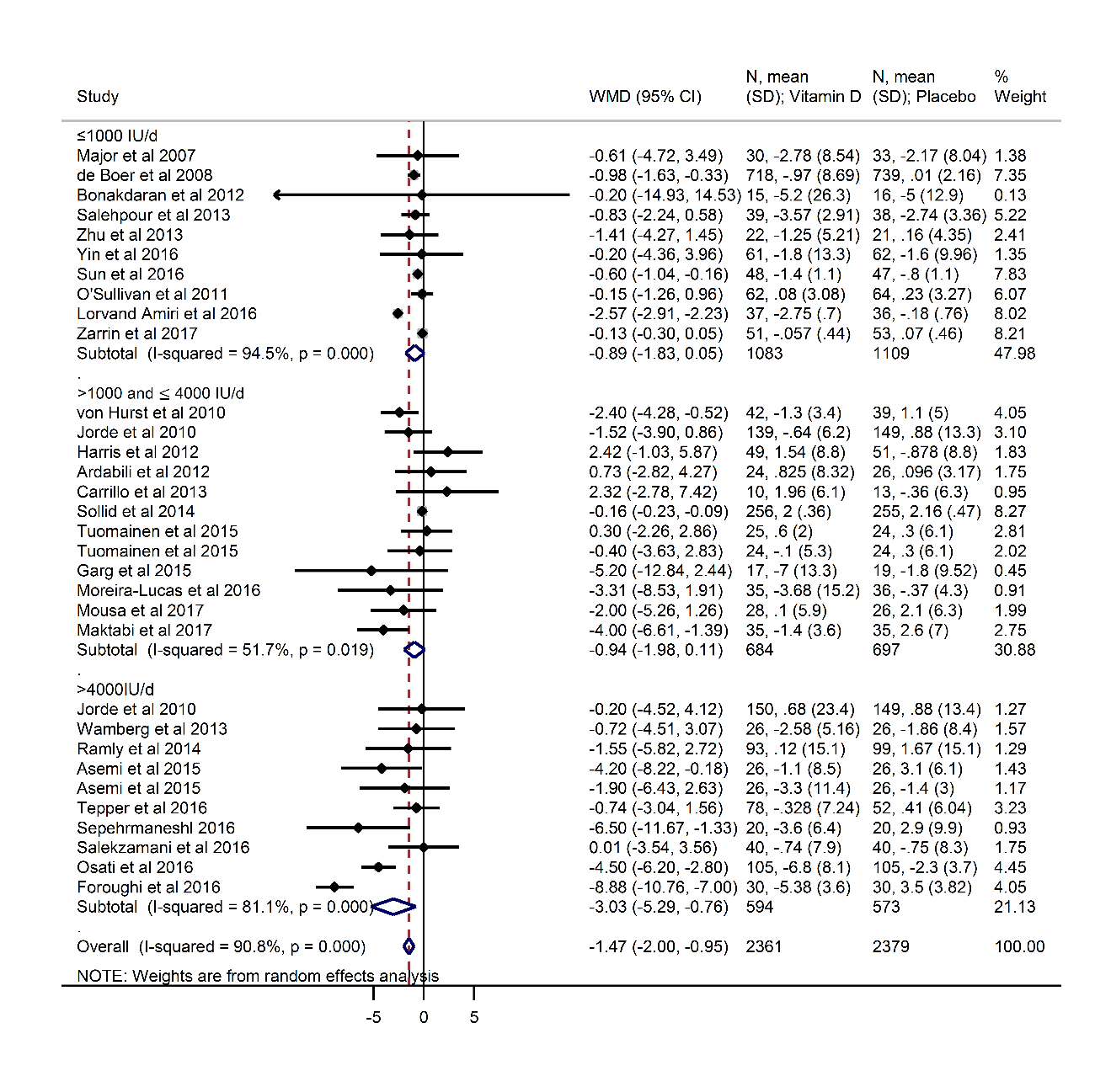


**Supplementary Figure 6. Meta-analysis of vitamin D supplementation on fasting glucose stratified by dose.**


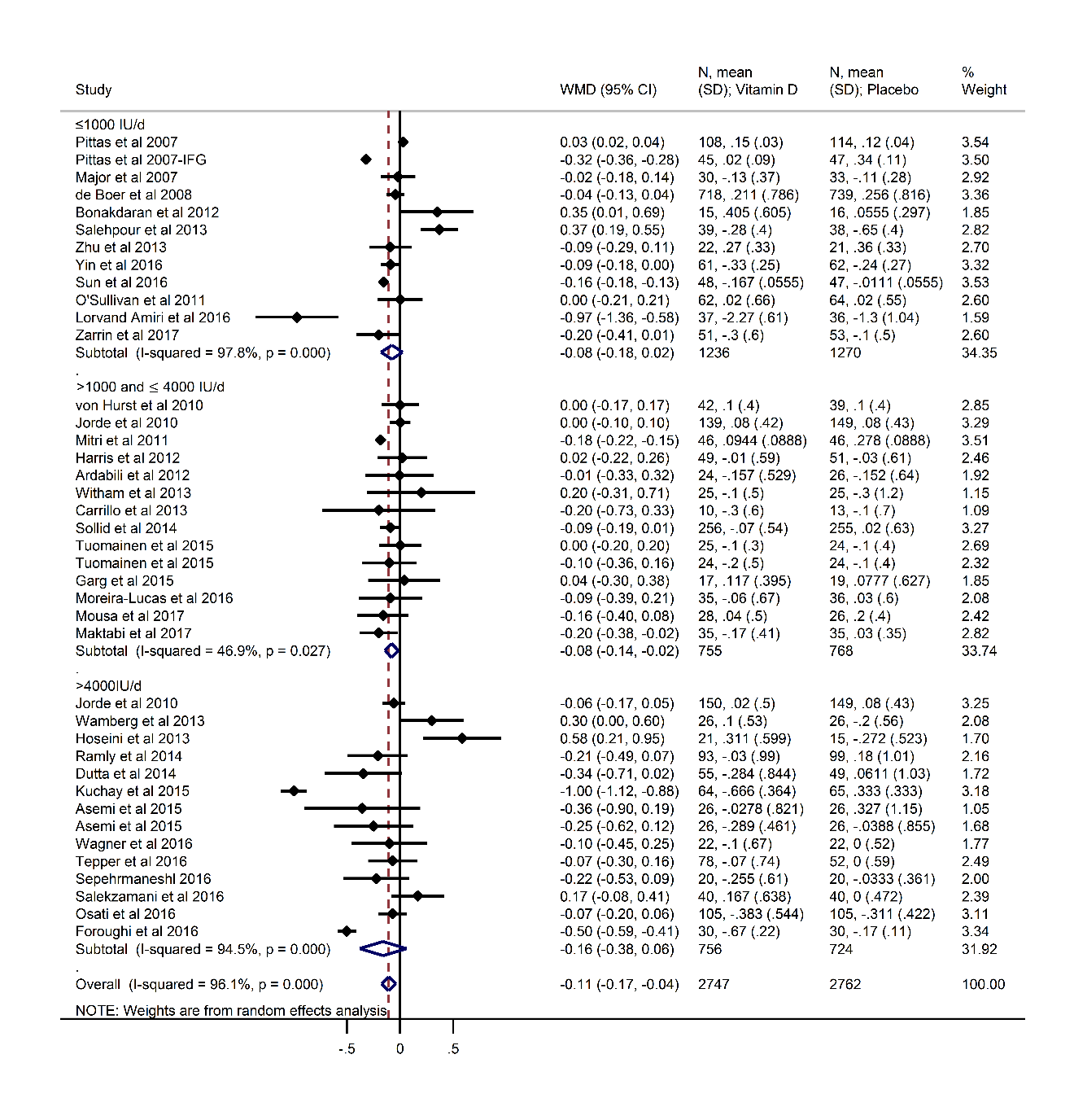


**Supplementary Figure 7. Meta-analysis of vitamin D supplementation on HOMA-IR stratified by dose.**


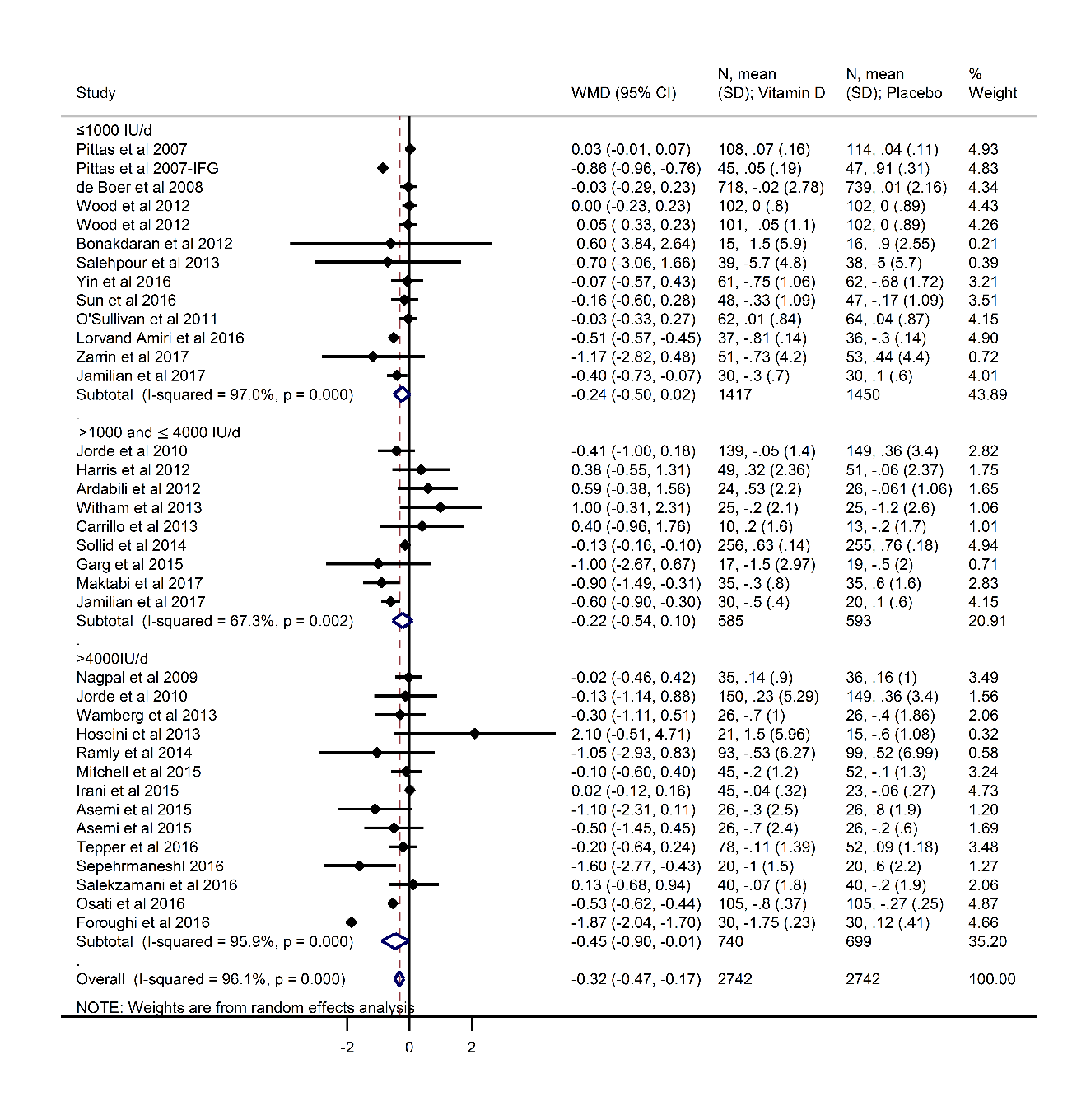


**Supplementary Figure 8.** Dose-response association between dose of vitamin D supplementation and change in serum 25(OH)D levels (A), fasting glucose (B), insulin (C), HbA1c (D), QUICKI (E), and HOMA-IR (F) by using restricted cubic spline curves. The red lines and the gray shaded regions indicated the estimated value and 95% confidence interval


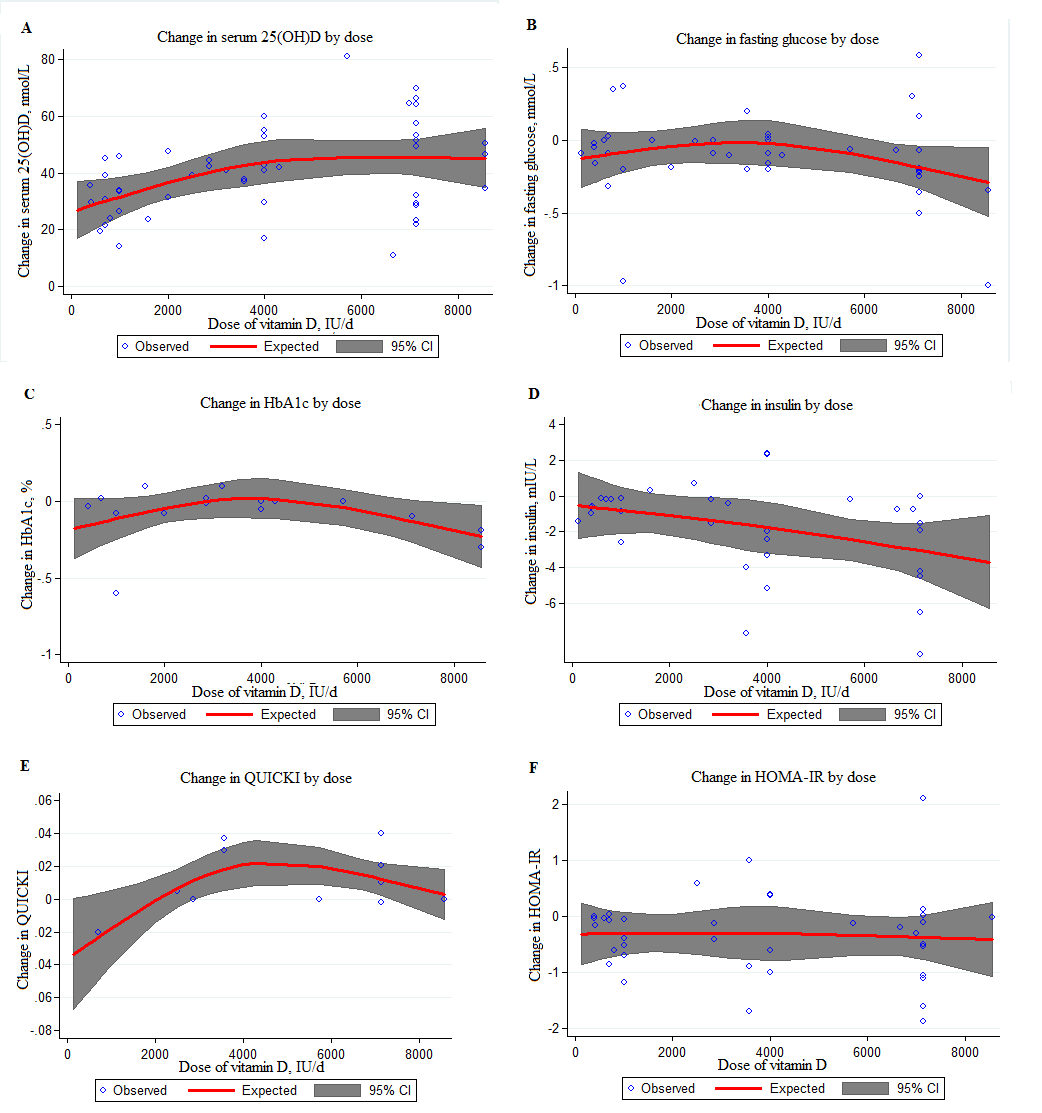


**Supplementary Figure 9.** Dose-response association between duration of vitamin D supplementation and change in serum 25(OH)D levels (A), fasting glucose (B), insulin (C), HbA1c (D), QUICKI (E), and HOMA-IR (F) by using restricted cubic spline curves. The red lines and the gray shaded regions indicated the estimated value and 95% confidence interval.


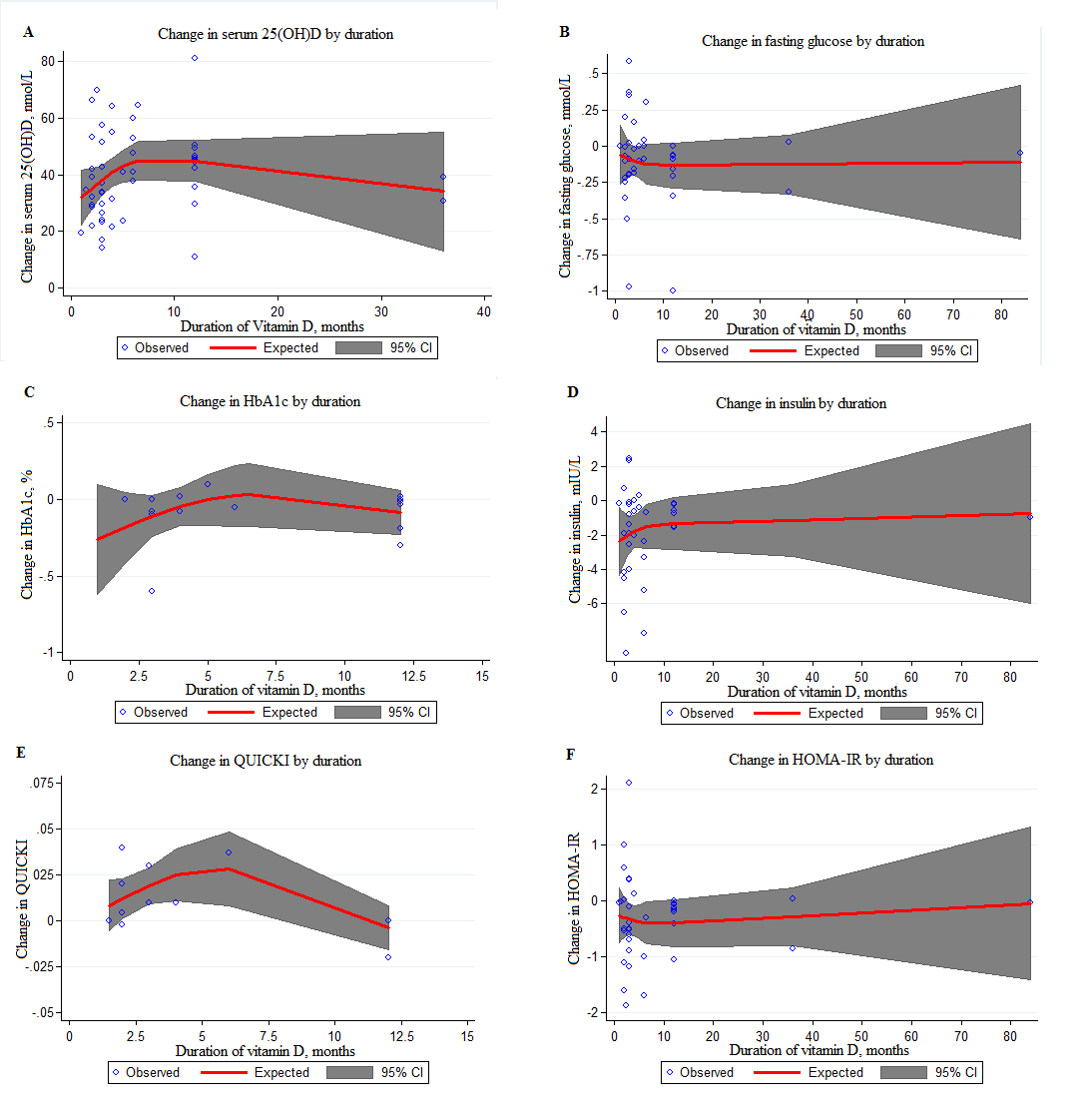


**Supplementary Figure 10** Meta-analysis of vitamin D supplementation on prediabetes progression to diabetes and its reversal to normoglycemia among participants with prediabetes.


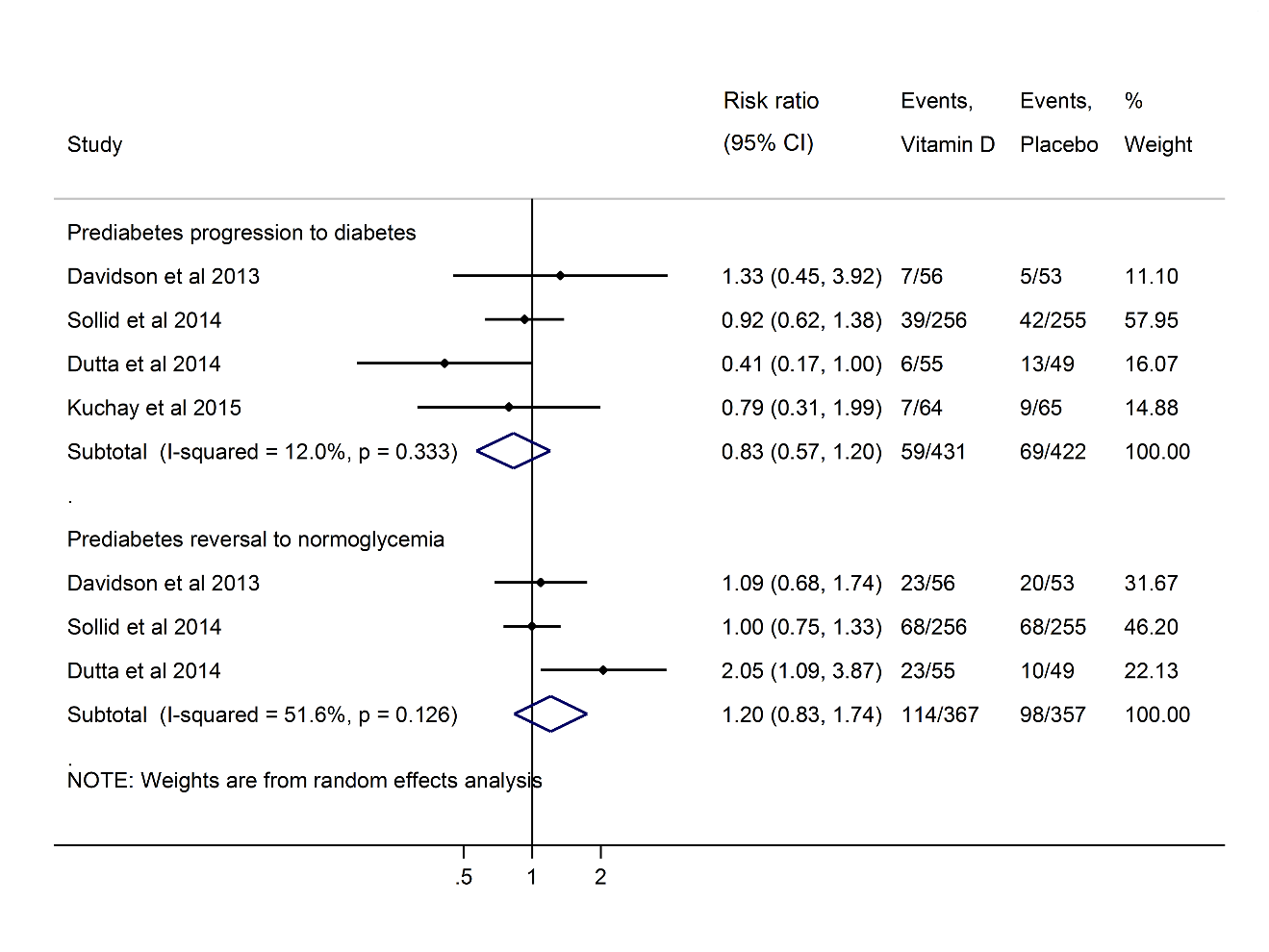


**Supplementary Table 1.** Characteristics of included studies

| **Study** | **Country** | **Subjects** | **N** | **Mean age (years)** | **Female**  **(%)** | **Mean or median BMI (kg/m^2^)** | **Mean or median baseline 25OHD (nmol/L)** | **Interventions** | **Controls** | **Duration** | **Outcomes** |
| --- | --- | --- | --- | --- | --- | --- | --- | --- | --- | --- | --- |
| Pittas et al 2007[[1](#_ENREF_1)] | US | Participants were healthy, ambulatory adults aged ≥65 years | 314 | 71.2 | 57.6 | 26.7 | 76 | Vitamin D3 (700 IU/d) + calcium (500 mg/d) | Placebo | 3 years | 25(OH)D, FG, HOMA-IR, diabetes |
| Major et al 2007 [[2](#_ENREF_2)] | Canada | Healthy, overweight or obese women | 63 | 42.6 | 100 | 31.8 | NR | Vitamin D  (400 IU/d) + Calcium (1,200 mg/d) | placebo | 15 weeks | FG, FI, 2h glucose |
| de Boer et al 2008 [[3](#_ENREF_3)] | US | Healthy postmenopausal women without self-reported diabetes | 33951 | 62 | 100% | NR | NR | Vitamin D3 (1000 mg/d) calcium+ (400 IU/d) | Placebo | 7 years | Diabetes, FG, insulin, HOMA-IR |
| Avenell et al 2009 [[4](#_ENREF_4)] | UK | Post hoc analysis of RECORD trial | 5292 | 77 | 84.7 | NR | NR | Vitamin D3 (800 IU/d) + calcium (100 mg/d) | Placebo + calcium (100 mg/d) | 2 years | Diabetes |
| Nagpal et al 2009 [[5](#_ENREF_5)] | India | Healthy centrally obese men | 71 | 43.7 | 0 | 26.4 | 33.2 | Vitamin D3 (three doses of 120,000 IU) | Placebo | 6 weeks | 25(OH)D, HOMA-IR, HOMA-2IR, QUICKI, HOMA-%B, HOMA2-%B |
| Jorde et al 2010 [[6](#_ENREF_6)] | Norway | Overweight or obese subjects | 438 | 47.5 | 64.2 | 34.7 | 58 | Vitamin D3 (40,000 IU/ week; 20,000 IU/week) + calcium (500 mg/d) | Placebo + calcium (500 mg/d) | 12 months | 25(OH)D, FG, FI, 2h glucose, 2h insulin, Hba1c, HOMA-IR, QUICK |
| von Hurst et al 2010 [[7](#_ENREF_7)] | New Zealand | Patients with insulin resistant and vitamin D deficiency (<50 nmol/l) | 81 | 41.6 | NR | 27.5 | 20 (Median) | Vitamin D3 (4,000 IU/d) | Placebo | 6 months | 25(OH)D, HOMA2-IR, FI, FG, HOMA2%S, HOMA2%B |
| Mitri et al 2011 [[8](#_ENREF_8)] | US | Patients with glucose intolerance or early diabetes | 92 | 57 | 51 | 32 | 61.2 | Vitamin D3 (2,000 IU/d) ±calcium (800 mg/d) | Placebo ±calcium (800 mg/d) | 16 weeks | 25(OH)D, Hba1c, FG, 2h glucose |
| O'Sullivan et al 2011 [[9](#_ENREF_9)] | Ireland | healthy volunteers | 135 | 35 | 55.6 | 24.2 | 56.6 | Vitamin D3 (15 µg/d) | placebo | 4 weeks | 25(OH)D, FG, FI, HOMA-IR |
| Ardabili et al 2012 [[10](#_ENREF_10)] | Iran | Women with PCOS and vitamin D deficiency | 60 | 26.9 | 100 | 28.7 | 18.5 | Vitamin D3 (50,000 IU/20ds) | Placebo | 2 months | 25(OH)D, FG, Insulin, HOMA-IR, HOMA-S, HOMA-B, QUICKI |
| Bonakdaran et al 2012 [[11](#_ENREF_11)] | Iran | PCOS patients | 51 | 25.3 | 100 | 26.1 | 19.8 | Vitamin D3 (0.5 µg/d) | Placebo | 3 months | 25(OH)D, FG, Insulin, 2h glucose, HOMA-IR |
| Harris et al 2012 [[12](#_ENREF_12)] | USA | Overweight or obese African Americans with prediabetes or early diabetes | 89 | 56.6 | 50.6 | 32.2 | 38.9 | Vitamin D3 (4000 IU/d) + calcium (600 mg/d) | Placebo + calcium (600 mg/d) | 12 weeks | 25(OH)D, FI, FG, Hba1c, HOMA-IR, 2-h glucose, DI |
| Simha et al 2012[[13](#_ENREF_13)] | US | Healthy subjects with serum 25(OH)D<20 ng/mL | 12 | 28.4 | 33 | 24.2 | 36.2 | Vitamin D2 (50,000 IU/week) | placebo | 8 weeks | 25(OH)D |
| Wood et al 2012 [[14](#_ENREF_14)] | UK | Healthy postmenopausal women | 305 | 63.8 | 100 | 26.7 | 33.8 | Vitamin D3 (400 or 1000 IU/d) | placebo | 12 months | 25(OH)D, HOMA-IR |
| Carrillo et al 2013 [[15](#_ENREF_15)] | USA | Overweight and obese adults | 23 | 26.1 | 52.2 | 31.3 | 19.3 | Vitamin D3 (4000 IU/d) + calcium (500 mg/d) | Placebo + calcium (500 mg/d) | 12 weeks | 25(OH)D, FG,2h glucose, Insulin, HOMA-IR |
| Davidson et al 2013 [[16](#_ENREF_16)] | USA | Subjects with prediabetes and  25(OH)D < 30 ng/mL | 109 | 52.4 | 67.9 | 32.5 | 22 | Vitamin D (88,865 IU/d) | Placebo | 12 months | Diabetes |
| Hoseini et al 2013 [[17](#_ENREF_17)] | Iran | People with pre-diabetes | 45 | 47.4 | 71.1 | 29.4 | 52.5 | Vitamin D (50,000 IU/week) + calcium (500 mg/d) | Placebo + calcium (500 mg/d) | 12 weeks | 25(OH)D, HOMA-IR, HOMA-B, HbA1c, FG |
| Salehpour et al 2013 [[18](#_ENREF_18)] | Iran | healthy overweight or obese women | 77 | 38 | 100 | 29.9 | 41.8 | Vitamin D3 (1,000 IU/d) | placebo | 12 weeks | 25(OH)D, FG, 2h glucose, FI, HbA1c, HOMA-IR |
| Witham et al 2013 [[19](#_ENREF_19)] | UK | Female patients (≥18) with 25(OH)D<75 nmol/L | 50 | 40.6 | 100 | 26.8 | 27 | Vitamin D3 (100,000 IU once) | Placebo | 4, 8 weeks | 25(OH)D, FG, HOMA-IR |
| Wamberg et al 2013 [[20](#_ENREF_20)] | Denmark | Healthy adults with BMI > 30 kg/m2 and 25(OH)D <50 nmol/l | 52 | 40.3 | 71.1 | 35.6 | 34.5 | Vitamin D3 (7,000 IU/d) | Placebo | 26 weeks | 25(OH)D, glucose, insulin, HOMA-IR |
| Zhu et al 2013 [[21](#_ENREF_21)] | China | Healthy, overweight and obese adults with very-low calcium consumption | 43 | 20.2 | 91 | 26 | NR | Vitamin D (125 IU/d) + Calcium (600 mg/d) | None | 12 weeks | Glucose, insulin |
| Dutta et al 2014 [[22](#_ENREF_22)] | India | Prediabetes (IPD) with 25(OH)D <30 ng/ml | 170 | 47.4 | 59.4 | 26.2 | 24.3 | Vitamin D3 (60,000 IU/week for 8 weeks and then 60,000 IU/month) + calcium (500 mg/d) | Calcium (500 mg/d) | 12 months | 25(OH)D, Hba1c, FG, 2-h glucose, HOMA2-IR, HOMA2-B, QUICKI, Diabetes |
| Gagnon et al 2014 [[23](#_ENREF_23)] | Australian | Adults with prediabetes or an AUSDRISK score≥15 | 80 | 54 | 69 | 31.5 | 45 | Vitamin D3 (2,000 IU/d) + calcium (1,200 mg/d) | Placebo | 6 months | 25(OH)D, HOMA-%S, DI |
| Madar et al 2014[[24](#_ENREF_24)] | Norway | Healthy adults | 251 | 37.3 | 72.5 | 27.4 | 28.9 | Vitamin D3 (10 µg/d or 25 µg/d) | Placebo | 16 weeks | 25(OH)D, Hba1c |
| Ramly et al 2014 [[25](#_ENREF_25)] | Malaysia | Premenopausal women with vitamin D deficiency | 192 | 42.7 | 100 | 27.2 | 30.1 | Vitamin D3 (50,000 IU /week) | placebo | 6 months, 12 months | 25(OH)D, FG, FI, HOMA-IR |
| Sollid et al 2014 [[26](#_ENREF_26)] | Norway | IFG and/or IGT patients aged 21-80 years | 511 | 62.1 | 38.6 | 29.9 | 59.9 | Vitamin D3 (20,000 IU/week) | placebo | 12 months | 25(OH)D, Hba1c, HOMA-IR, QUICKI, FG, 2Hsg-OGTT, FI, 2Hsi-OGTT |
| Asemi et al 2015 [[27](#_ENREF_27)] | Iran | Overweight and obese vitamin D deficient women with PCOS | 104 | 24.9 |  | 28.1 | 13.6 | Vitamin D3 (50,000 IU/week) ± calcium (1000 mg/d) | Placebo ± calcium (1000 mg/d) | 8 weeks | 25(OH)D, FG, Insulin, HOMA-IR, QUICKI |
| Foroozanfard et al 2015 [[28](#_ENREF_28)] | Iran | Overweight and vitamin  D-deficient women with polycystic ovary syndrome | 104 | NR | NR | NR | NR | Vitamin D3  (50,000 IU/ week) with or without calcium (1,000 mg/d) | Placebo with or without calcium (1,000 mg/d) | 8 weeks | HOMA-B |
| Kuchay et al 2015 [[29](#_ENREF_29)] | India | Prediabetes on the basis of elevated A1C  levels, FPG and 2‑h plasma glucose during OGTT | 129 | 48.05 |  | 25.5 | 48 | Vitamin D3 (60,000 IU weekly for 4 weeks and then 60,000 IU/month) | no Vitamin D3 | 12 months | 25(OH)D, FG, 2h glucose, Hba1c, Diabetes, |
| Mitchell et al 2015 [[30](#_ENREF_30)] | US | Healthy people with low total 25(OH)D | 90 | 28.5 | 61.1 | 25.6 | 45 | Vitamin D2 (50,000 IU/wk) + calcium (1000-1500 mg/d) | Placebo+calcium (1000-1500 mg/d) | 12 weeks | 25(OH)D, HOMA-IR |
| Irani et al 2015 [[31](#_ENREF_31)] | US | Vitamin D deficient women with PCOS | 68 | 30 | 100 | 29 | 41.8 | Vitamin D3 (50,000 IU/week) | Placebo | 8 weeks | 25(OH)D, HOMA-IR, HOMA%B |
| Garg et al 2015 [[32](#_ENREF_32)] | India | PCOS women | 36 | 22.4 | 100 | 26.7 | 18 | Vitamin D3 (4000 IU/d) + metformin (500 mg/d) | Placebo + metformin (500 mg/d) | 6 months | 25(OH)D, FG, FI, HOMA-IR, DI |
| Tuomainen et al 2015 [[33](#_ENREF_33)] | Eastern Finland | Patients with disturbed glucose homeostasis and serum 25(OH)D3 <75 nmol/L. | 73 | Median 65.7 | NR | 29.4 | 57 | Vitamin D3 (1,600 IU/d or 3,200 IU/d) | Placebo | 5 months | 25(OH)D, hba1c, FG, FI, HOMA IR, HOMA2%IR/B |
| Foroughi et al 2016 [[34](#_ENREF_34)] | Iran | Patients with non-alcoholic fatty liver disease | 60 | 48.5 | 51.7 | NR | 48 | Vitamin D3  (50,000 IU/ week) | Placebo | 10 weeks | 25(OH)D, FG, HOMA-IR, HOMA-B |
| Lorvand Amiri et al 2016 [[35](#_ENREF_35)] | Iran | Non-alcoholic fatty liver patients following  an energy-restricted diet | 73 | 42 | 38 | 31 | 10 | Vitamin D3  (1,000 IU/d) | Placebo | 12 weeks | 25(OH)D, FG, insulin, HOMA-IR |
| Moreira-Lucas et al 2016 [[36](#_ENREF_36)] | Canada | Subjects with serum 25(OH)D ≤65 nmol/L, IFG and elevated glycated hemoglobin | 71 | 47.3 | 53.5 | 30.9 | 47.8 | Vitamin D3 (28,000 IU/week) | Placebo | 24 weeks | FG, 2h glucose, FI, 2h insulin, HbA1c%, HOMA2%S, HOMA2%B, DI |
| Wagner et al 2016 [[37](#_ENREF_37)] | Sweden | Abnormal glucose tolerance patients with 25(OH)D<75 nmol/L | 44 | 67.3 | 46.5 | 28.5 | 47 | Vitamin D3 (30,000 IU/week) | placebo | 8 weeks | 25(OH)D; HbA1c; FPG; 2-h OGTT |
| Yin et al 2016 [[38](#_ENREF_38)] | China | Subjects with metabolic syndrome and hypovitaminosis D, otherwise healthy | 126 | 49.5 | 46 | 27.1 | 36.2 | Vitamin D3 (700 IU/d) | placebo | 12 months | FG, HOMA-IR, QUICKI, 25(OH)D |
| Sun et al 2016 [[39](#_ENREF_39)] | Japan | Healthy adults | 95 | 43 | 64.2 | 22.1 | 32.4 | Vitamin D3 (420 IU/d) + calcium | Placebo + calcium | 12 months | 25(OH)D, FG, FI, HOMA-IR, Hba1c |
| Sepehrmanesh et al 2016 [[40](#_ENREF_40)] | Iran | Patients with major depressive disorder | 40 | 36.3 | 85 | 26.4 | 28.4 | Vitamin D (50,000 IU/week) | placebo | 8 weeks | 25(OH)D, FI, FPG, HOMA-IR, HOMA-B QUICKI |
| Salekzamani et al 2016 [[41](#_ENREF_41)] | Iran | Patients with metabolic syndrome | 80 | 40.5 | 51 | NR | 20 | Vitamin D3 (50,000 IU/week) vitamin D | placebo | 16 weeks | 25(OH)D, FG, Insulin, HOMA-IR, QUICKI |
| Tepper et al 2016 [[42](#_ENREF_42)] | Israel | Subjects aged 20–65 years, with serum 25(OH)D <50nmol/l | 130 | 47.5 | 0% | 27.8 | 38.9 | Vitamin D3 (100,000 IU/ bi-months) | placebo | 6, 12 months | 25(OH)D, FG; FI; HOMA-IR; HOMA-β |
| Osati et al 2016 [[43](#_ENREF_43)] | Iran | healthy overweight people | 210 | 38 | 76.7 | 31.5 | 34.5 | Vitamin D (50,000 IU/week) | placebo | 8 weeks | FG, 2h glucose, FI, 2h insulin, HOMA-IR, HOMA-β%, HOMA-S% |
| Jamilian et al 2017 [[44](#_ENREF_44)] | Iran | Women with PCOS | 90 | 26.3 | 100 | 31.3 | 31.7 | Vitamin D (4,000 IU/d)  Vitamin D  (1,000 IU/d) | Placebo | 12 weeks | 25(OH)D, HOMA-IR |
| Mousa et al 2017 [[45](#_ENREF_45)] | Australia | Vitamin D–deficient, overweight or obese adults | 54 | Median： 30 | 54 | Mediun:30.1 | 32.7 | Vitamin D3 (100,000 IU followed by 4,000 IU/d) | Placebo | 16 weeks | 25(OH)D, FG, FI, |
| Maktabi et al 2017 [[46](#_ENREF_46)] | Iran | Polycystic ovary syndrome women | 70 | 22.5 | 100 | 23.4 | 34 | Vitamin D3  (50,000 IU/2 weeks) | Placebo | 12 weeks | 25(OH)D, FG, insulin, HOMA-IR, HOMA-B, QUICKI |
| Zarrin et al 2017 [[47](#_ENREF_47)] | Iran | Adults with Prediabetes | 120 | 48 | 51 | 29 | 55 | Vitamin D (1,000 IU/d) | Placebo | 3 months | 25(OH)D, HbA1c, FG, insulin, HOMA-IR |

25(OH)D, 25-hydroxyvitamin D; FG, fasting glucose; FI, fasting insulin; HOMA-IR, homeostatic model assessment-insulin resistance; QUICKI, quantitative insulin sensitivity check index; WMD, weighted mean difference; 2h glucose, 2-hour plasma glucose; 2h insulin, 2-hour plasma insulin; DI, disposition index; IFG, impaired fasting glucose.

**Supplementary Table 2.** Meta-analysis of vitamin D supplementation on indexes of glucose and insulin homeostasis stratified by duration

|  | Overall | | | < 3 months | | | ≥ 3 and < 12 months | | | ≥ 12 months | | | P for trend |
| --- | --- | --- | --- | --- | --- | --- | --- | --- | --- | --- | --- | --- | --- |
|  | n/N | WMD | I^2^ (%) | n/N | WMD | I^2^ (%) | n/N | WMD | I^2^ (%) | n/N | WMD | I^2^ (%) |  |
| 25(OH)D | 46/4992 | 40.14  (37.07, 43.22) | 97.8 | 11/785 | 39.74  (27.86, 51.62) | 98.1 | 22/1733 | 38.27  (34.57,41.96) | 94.3 | 13/2592 | 41.96  (36.99, 46.93) | 98.2 | 0.70 |
| HbA1c | 16/2298 | -0.04  (-0.07, 0.00) | 86.2 | 1/44 | 0.00  (-0.18, 0.18) | NA | 9/828 | -0.04  (-0.11, 0.04) | 86.5 | 6/1426 | -0.05  (-0.10, 0.01) | 83.4 | 0.71 |
| Fasting glucose | 40/5509 | -0.11  (-0.17, -0.04) | 96.1 | 9/684 | -0.16  (-0.35, 0.04) | 83.1 | 19/1183 | -0.02  (-0.12, 0.09) | 82.2 | 12/3642 | -0.19  (-0.30, -0.08) | 98.5 | 0.55 |
| Insulin | 32/4740 | -1.47  (-2.00, -0.95) | 90.8 | 7/590 | -3.62  (-6.75, -0.48) | 91.7 | 17/1055 | -1.12  (-2.18, -0.06) | 91.1 | 8/3095 | -0.49  (-0.86, -0.13) | 38.2 | 0.08 |
| 2h glucose | 15/1929 | -0.06  (-0.47, 0.35) | 88.4 | 1/44 | 0.40  (-0.86, 1.66) | NA | 9/554 | 0.08  (-0.57, 0.72) | 88.4 | 5/1331 | -0.28  (-0.85, 0.30) | 89.8 | 0.35 |
| 2h insulin | 5/1379 | -2.17  (-15.6, 11.25) | 93.4 | 1/210 | -26.60  (-34.77, -18.43) | NA | 1/71 | 7.86  (-12.09, 27.81) | NA | 3/1098 | 5.66  (4.89, 6.43) | 0 | 0.01 |
| **Insulin sensitivity** | | | | | | | | | | | | | |
| HOMA-IR | 36/5484 | -0.32  (-0.47, -0.17) | 96.1 | 10/779 | -0.43  (-0.95, 0.09) | 97.3 | 14/889 | -0.43  (-0.60, -0.26) | 26.3 | 12/3816 | -0.20  (-0.36, -0.03) | 95.8 | 0.15 |
| QUICKI | 12/1740 | 0.00  (-0.00, 0.01) | 72.1 | 5/265 | 0.01  (-0.00, 0.03) | 77.9 | 2/150 | 0.02  (0.00, 0.04) | 60.2 | 5/1325 | -0.00  (-0.00, 0.00) | 0 | 0.05 |
| HOMA2-IR | 5/353 | -0.14  (-0.31, 0.04) | 46.6 | 1/71 | 0.04  (-0.17, 0.25) | NA | 3/178 | -0.21  (-0.46, 0.040 | 42.1 | 1/104 | -0.18  (-0.47, 0.11) | NA | 0.43 |
| HOMA2-%S | 5/329 | 0.57  (-3.24, 4.37) | 22.6 | NA | NA | NA | 5/329 | 0.57  (-3.24, 4.37) | 22.6 | NA | NA | NA | NA |
| **Beta cell function** | | | | | | | | | | | | | |
| HOMA-B | 6/404 | -10.69  (-19.10, -2.29) | 87.1 | 4/204 | -9.79  (-20.24, 0.66) | 92.1 | 1/70 | -14.80  (-24.76, -4.84) | NA | 1/130 | -11.23  (-39.86, 17.40) | NA | 0.93 |
| HOMA-%B | 2/139 | 4.87  (-44.59, 54.34) | 84.5 | 2/139 | 4.87  (-44.59, 54.34) | 84.5 | NA | NA | NA | NA | NA | NA | NA |
| HOMA2-%B | 5/320 | 1.57  (-3.98, 7.12) | 0 | 1/71 | 12.03  (-4.70, 28.76) | NA | 4/249 | 0.28  (-5.61, 6.16) | 0 | NA | NA | NA | 0.29 |
| Disposition index | 3/287 | -0.05  (-0.39, 0.29) | 1.9 | NA | NA | NA | 3/287 | -0.05  (-0.39, 0.29) | 1.9 | NA | NA | NA | NA |

n/N, number of studies/number of participants; 25(OH)D, 25-hydroxyvitamin D; HOMA-IR, homeostatic model assessment-insulin resistance; QUICKI, quantitative insulin sensitivity check index; WMD, weighted mean difference; 2h glucose, 2-hour plasma glucose; 2h insulin, 2-hour plasma insulin; NA, not applicable.

**References**

[1] Pittas AG, Harris SS, Stark PC, Dawson-Hughes B. The effects of calcium and vitamin D supplementation on blood glucose and markers of inflammation in nondiabetic adults. Diabetes care. 2007;30:980-6.

[2] Major GC, Alarie F, Dore J, Phouttama S, Tremblay A. Supplementation with calcium + vitamin D enhances the beneficial effect of weight loss on plasma lipid and lipoprotein concentrations. The American journal of clinical nutrition. 2007;85:54-9.

[3] Boer IH, Tinker LF, Connelly S, Curb JD, Howard BV, Kestenbaum B, et al. Calcium plus vitamin D supplementation and the risk of incident diabetes in the women's health initiative. Diabetes care. 2008;31:701-7.

[4] Avenell A, Cook JA, MacLennan GS, McPherson GC. Vitamin D supplementation and type 2 diabetes: a substudy of a randomised placebo-controlled trial in older people (RECORD trial, ISRCTN 51647438). Age Ageing. 2009;38:606-9.

[5] Nagpal J, Pande JN, Bhartia A. A double-blind, randomized, placebo-controlled trial of the short-term effect of vitamin D3 supplementation on insulin sensitivity in apparently healthy, middle-aged, centrally obese men. Diabetic medicine : a journal of the British Diabetic Association. 2009;26:19-27.

[6] Jorde R, Sneve M, Torjesen P, Figenschau Y. No improvement in cardiovascular risk factors in overweight and obese subjects after supplementation with vitamin D3 for 1 year. J Intern Med. 2010;267:462-72.

[7] von Hurst PR, Stonehouse W, Coad J. Vitamin D supplementation reduces insulin resistance in South Asian women living in New Zealand who are insulin resistant and vitamin D deficient - a randomised, placebo-controlled trial. Br J Nutr. 2010;103:549-55.

[8] Mitri J, Dawson-Hughes B, Hu FB, Pittas AG. Effects of vitamin D and calcium supplementation on pancreatic beta cell function, insulin sensitivity, and glycemia in adults at high risk of diabetes: the Calcium and Vitamin D for Diabetes Mellitus (CaDDM) randomized controlled trial. The American journal of clinical nutrition. 2011;94:486-94.

[9] O'Sullivan A, Gibney MJ, Connor AO, Mion B, Kaluskar S, Cashman KD, et al. Biochemical and metabolomic phenotyping in the identification of a vitamin D responsive metabotype for markers of the metabolic syndrome. Molecular nutrition & food research. 2011;55:679-90.

[10] Ardabili HR, Gargari BP, Farzadi L. Vitamin D supplementation has no effect on insulin resistance assessment in women with polycystic ovary syndrome and vitamin D deficiency. Nutrition research (New York, NY). 2012;32:195-201.

[11] Bonakdaran S, Mazloom Khorasani Z, Davachi B, Mazloom Khorasani J. The effects of calcitriol on improvement of insulin resistance, ovulation and comparison with metformin therapy in PCOS patients: a randomized placebo- controlled clinical trial. Iran J Reprod Med. 2012;10:465-72.

[12] Harris SS, Pittas AG, Palermo NJ. A randomized, placebo-controlled trial of vitamin D supplementation to improve glycaemia in overweight and obese African Americans. Diabetes Obes Metab. 2012;14:789-94.

[13] Simha V, Mahmood M, Ansari M, Spellman CW, Shah P. Effect of vitamin D replacement on insulin sensitivity in subjects with vitamin D deficiency. Journal of investigative medicine : the official publication of the American Federation for Clinical Research. 2012;60:1214-8.

[14] Wood AD, Secombes KR, Thies F, Aucott L, Black AJ, Mavroeidi A, et al. Vitamin D3 supplementation has no effect on conventional cardiovascular risk factors: a parallel-group, double-blind, placebo-controlled RCT. J Clin Endocrinol Metab. 2012;97:3557-68.

[15] Carrillo AE, Flynn MG, Pinkston C, Markofski MM, Jiang Y, Donkin SS, et al. Impact of vitamin D supplementation during a resistance training intervention on body composition, muscle function, and glucose tolerance in overweight and obese adults. Clinical Nutrition. 2013;32:375-81.

[16] Davidson MB, Duran P, Lee ML, Friedman TC. High-dose vitamin D supplementation in people with prediabetes and hypovitaminosis D. Diabetes Care. 2013;36:260-6.

[17] Hoseini SA, Aminorroaya A, Iraj B, Amini M. The effects of oral vitamin D on insulin resistance in pre-diabetic patients. Journal of research in medical sciences : the official journal of Isfahan University of Medical Sciences. 2013;18:47-51.

[18] Salehpour A, Shidfar F, Hosseinpanah F, Vafa M, Razaghi M, Amiri F. Does vitamin D3 supplementation improve glucose homeostasis in overweight or obese women? A double-blind, randomized, placebo-controlled clinical trial. Diabetic medicine : a journal of the British Diabetic Association. 2013;30:1477-81.

[19] Witham MD, Adams F, Kabir G, Kennedy G, Belch JJF, Khan F. Effect of short-term vitamin D supplementation on markers of vascular health in South Asian women living in the UK - A randomised controlled trial. Atherosclerosis. 2013;230:293-9.

[20] Wamberg L, Kampmann U, Stodkilde-Jorgensen H, Rejnmark L, Pedersen SB, Richelsen B. Effects of vitamin D supplementation on body fat accumulation, inflammation, and metabolic risk factors in obese adults with low vitamin D levels - results from a randomized trial. European journal of internal medicine. 2013;24:644-9.

[21] Zhu W, Cai D, Wang Y, Lin N, Hu Q, Qi Y, et al. Calcium plus vitamin D3 supplementation facilitated fat loss in overweight and obese college students with very-low calcium consumption: a randomized controlled trial. Nutr J. 2013;12:8.

[22] Dutta D, Mondal SA, Choudhuri S, Maisnam I, Hasanoor Reza AH, Bhattacharya B, et al. Vitamin-D supplementation in prediabetes reduced progression to type 2 diabetes and was associated with decreased insulin resistance and systemic inflammation: an open label randomized prospective study from Eastern India. Diabetes Res Clin Pract. 2014;103:e18-23.

[23] Gagnon C, Daly RM, Carpentier A, Lu ZX, Shore-Lorenti C, Sikaris K, et al. Effects of combined calcium and vitamin D supplementation on insulin secretion, insulin sensitivity and beta-cell function in multi-ethnic vitamin D-deficient adults at risk for type 2 diabetes: a pilot randomized, placebo-controlled trial. PLoS One. 2014;9:e109607.

[24] Madar AA, Knutsen KV, Stene LC, Brekke M, Meyer HE, Lagerlov P. Effect of vitamin D3 supplementation on glycated hemoglobin (HbA1c), fructosamine, serum lipids, and body mass index: a randomized, double-blinded, placebo-controlled trial among healthy immigrants living in Norway. BMJ open diabetes research & care. 2014;2:e000026.

[25] Ramly M, Ming MF, Chinna K, Suboh S, Pendek R. Effect of vitamin D supplementation on cardiometabolic risks and health-related quality of life among urban premenopausal women in a tropical country--a randomized controlled trial. PloS one. 2014;9:e110476.

[26] Sollid ST, Hutchinson MY, Fuskevag OM, Figenschau Y, Joakimsen RM, Schirmer H, et al. No effect of high-dose vitamin D supplementation on glycemic status or cardiovascular risk factors in subjects with prediabetes. Diabetes Care. 2014;37:2123-31.

[27] Asemi Z, Foroozanfard F, Hashemi T, Bahmani F, Jamilian M, Esmaillzadeh A. Calcium plus vitamin D supplementation affects glucose metabolism and lipid concentrations in overweight and obese vitamin D deficient women with polycystic ovary syndrome. Clin Nutr. 2015;34:586-92.

[28] Foroozanfard F, Jamilian M, Bahmani F, Talaee R, Talaee N, Hashemi T, et al. Calcium plus vitamin D supplementation influences biomarkers of inflammation and oxidative stress in overweight and vitamin D-deficient women with polycystic ovary syndrome: a randomized double-blind placebo-controlled clinical trial. Clin Endocrinol (Oxf). 2015;83:888-94.

[29] Kuchay MS, Laway BA, Bashir MI, Wani AI, Misgar RA, Shah ZA. Effect of Vitamin D supplementation on glycemic parameters and progression of prediabetes to diabetes: A 1-year, open-label randomized study. Indian journal of endocrinology and metabolism. 2015;19:387-92.

[30] Mitchell DM, Leder BZ, Cagliero E, Mendoza N, Henao MP, Hayden DL, et al. Insulin secretion and sensitivity in healthy adults with low vitamin D are not affected by high-dose ergocalciferol administration: a randomized controlled trial. The American journal of clinical nutrition. 2015;102:385-92.

[31] Irani M, Seifer DB, Grazi RV, Julka N, Bhatt D, Kalgi B, et al. Vitamin D Supplementation Decreases TGF-beta1 Bioavailability in PCOS: A Randomized Placebo-Controlled Trial. J Clin Endocrinol Metab. 2015;100:4307-14.

[32] Garg G, Kachhawa G, Ramot R, Khadgawat R, Tandon N, Sreenivas V, et al. Effect of vitamin D supplementation on insulin kinetics and cardiovascular risk factors in polycystic ovarian syndrome: a pilot study. Endocr Connect. 2015;4:108-16.

[33] Tuomainen TP, Virtanen JK, Voutilainen S, Nurmi T, Mursu J, de Mello VD, et al. Glucose Metabolism Effects of Vitamin D in Prediabetes: The VitDmet Randomized Placebo-Controlled Supplementation Study. Journal of diabetes research. 2015;2015:672653.

[34] Foroughi M, Maghsoudi Z, Askari G. The effect of vitamin D supplementation on blood sugar and different indices of insulin resistance in patients with non-alcoholic fatty liver disease (NAFLD). Iranian journal of nursing and midwifery research. 2016;21:100-4.

[35] Lorvand Amiri H, Agah S, Tolouei Azar J, Hosseini S, Shidfar F, Mousavi SN. Effect of daily calcitriol supplementation with and without calcium on disease regression in non-alcoholic fatty liver patients following an energy-restricted diet: Randomized, controlled, double-blind trial. Clin Nutr. 2016.

[36] Moreira-Lucas TS, Duncan AM, Rabasa-Lhoret R, Vieth R, Gibbs AL, Badawi A, et al. Effect of vitamin D supplementation on oral glucose tolerance in individuals with low vitamin D status and increased risk for developing type 2 diabetes (EVIDENCE): A double-blind, randomized, placebo-controlled clinical trial. Diabetes Obes Metab. 2016;19:133-41.

[37] Wagner H, Alvarsson M, Mannheimer B, Degerblad M, Ostenson CG. No Effect of High-Dose Vitamin D Treatment on beta-Cell Function, Insulin Sensitivity, or Glucose Homeostasis in Subjects With Abnormal Glucose Tolerance: A Randomized Clinical Trial. Diabetes Care. 2016;39:345-52.

[38] Yin X, Yan L, Lu Y, Jiang Q, Pu Y, Sun Q. Correction of hypovitaminosis D does not improve the metabolic syndrome risk profile in a Chinese population: a randomized controlled trial for 1 year. Asia Pac J Clin Nutr. 2016;25:71-7.

[39] Sun X, Cao ZB, Tanisawa K, Ito T, Oshima S, Higuchi M. Vitamin D supplementation reduces insulin resistance in Japanese adults: a secondary analysis of a double-blind, randomized, placebo-controlled trial. Nutrition Research. 2016;36:1121-9.

[40] Sepehrmanesh Z, Kolahdooz F, Abedi F, Mazroii N, Assarian A, Asemi Z, et al. Vitamin D Supplementation Affects the Beck Depression Inventory, Insulin Resistance, and Biomarkers of Oxidative Stress in Patients with Major Depressive Disorder: A Randomized, Controlled Clinical Trial. The Journal of nutrition. 2016;146:243-8.

[41] Salekzamani S, Mehralizadeh H, Ghezel A, Salekzamani Y, Jafarabadi MA, Bavil AS, et al. Effect of high-dose vitamin D supplementation on cardiometabolic risk factors in subjects with metabolic syndrome: a randomized controlled double-blind clinical trial. J Endocrinol Invest. 2016;39:1303-13.

[42] Tepper S, Shahar DR, Geva D, Ish-Shalom S. Differences in homeostatic model assessment (HOMA) values and insulin levels after vitamin D supplementation in healthy men: a double-blind randomized controlled trial. Diabetes Obes Metab. 2016;18:633-7.

[43] Osati S, Homayounfar R, Hajifaraji M. Metabolic effects of vitamin D supplementation in vitamin D deficient patients (a double-blind clinical trial). Diabetes & metabolic syndrome. 2016;10:S7-s10.

[44] Jamilian M, Foroozanfard F, Rahmani E, Talebi M, Bahmani F, Asemi Z. Effect of Two Different Doses of Vitamin D Supplementation on Metabolic Profiles of Insulin-Resistant Patients with Polycystic Ovary Syndrome. Nutrients. 2017;9.

[45] Mousa A, Naderpoor N, de Courten MP, Teede H, Kellow N, Walker K, et al. Vitamin D supplementation has no effect on insulin sensitivity or secretion in vitamin D-deficient, overweight or obese adults: a randomized placebo-controlled trial. The American journal of clinical nutrition. 2017;105:1372-81.

[46] Maktabi M, Chamani M, Asemi Z. The Effects of Vitamin D Supplementation on Metabolic Status of Patients with Polycystic Ovary Syndrome: A Randomized, Double-Blind, Placebo-Controlled Trial. Hormone and metabolic research = Hormon- und Stoffwechselforschung = Hormones et metabolisme. 2017;49:493-8.

[47] Zarrin R, Ayremlou P, Ghassemi F. The effect of vitamin D supplementation on the glycemic status and the percentage of body fat mass in adults with prediabetes: a randomized clinical trial. Iranian Red Crescent medical journal. 2017;19.
